# Synthesis, Characterization, and Antibacterial Activity of Ni-Substituted Krebs-type Sandwich-Tungstobismuthates Functionalized with Amino Acids

**DOI:** 10.1021/acs.inorgchem.3c00747

**Published:** 2023-06-07

**Authors:** Morteza Rafieeshad, Nadiia I. Gumerova, Elias Tanuhadi, Gerald Giester, Hana Čipčić-Paljetak, Donatella Verbanac, Annette Rompel

**Affiliations:** † Universität Wien, Fakultät für Chemie, Institut für Biophysikalische Chemie, Josef-Holaubek-Platz 2, Wien 1090, Austria; ‡ Fakultät für Geowissenschaften, Geographie und Astronomie, Institut für Mineralogie und Kristallographie, Universität Wien, Josef-Holaubek-Platz 2, Wien 1090, Austria; § Center for Translational and Clinical Research, School of Medicine, University of Zagreb, Šalata 2, Zagreb 10000, Croatia; ∥ Faculty of Pharmacy and Biochemistry, University of Zagreb, A. Kovačića 1, Zagreb 10000, Croatia

## Abstract

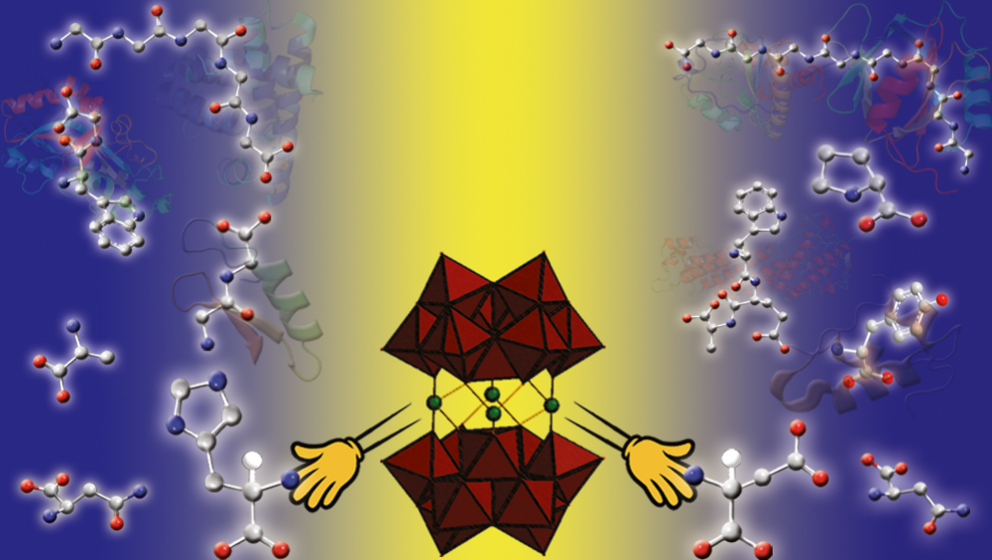

Four new Ni-substituted Krebs-type sandwich-tungstobismuthates, K_4_Ni_2_[{Ni(β-ala)(H_2_O)_2_}_2_{Ni(H_2_O)}_2_{Ni(H_2_O)(η^2^-β-ala)}_2_(*B*-β-BiW_9_O_33_)_2_]·49H_2_O **{(β-ala)**_**4**_**(Ni**_**3**_**)**_**2**_**(BiW**_**9**_**)**_**2**_**}**, K_3.5_Na_6.5_[{Ni(η^3^-L-asp)}_2_(WO_2_)_2_(*B*-β-BiW_9_O_33_)_2_]·36H_2_O·L-asp **{(L-asp)**_**2**_**(NiW)**_**2**_**(BiW**_**9**_**)**_**2**_**}**, K_4_Na_6_[{Ni(gly)(H_2_O)_2_}_2_(WO_2_)_2_(*B*-β-BiW_9_O_33_)_2_]·86H_2_O **{(gly)**_**2**_**(NiW)**_**2**_**(BiW**_**9**_**)**_**2**_**},** and K_2_Na_8_[{Ni(η^2^-serinol) (H_2_O)}_2_{Ni(H_2_O)_2_}_2_(*B*-β-BiW_9_O_33_)_2_]·42H_2_O **{(serinol)**_**2**_**Ni**_**4**_**(BiW**_**9**_**)**_**2**_**}** have been synthesized by one-pot solution methods. All compounds have been characterized in the solid state by single-crystal X-ray diffraction (SXRD), powder X-ray diffraction (PXRD), elemental and thermogravimetric analyses, and infrared spectroscopy (IR), as well as by UV–vis spectroscopy in solution. The antibacterial activity of all compounds was studied against four bacterial strains by the determination of the minimum inhibitory concentration (MIC). The results showed that only **{(β-ala)**_**4**_**(Ni**_**3**_**)**_**2**_**(BiW**_**9**_**)**_**2**_**}** demonstrates antibacterial activity (MIC is in the range from 8 to 256 μg/mL) compared to three other Ni-Krebs sandwiches.

## Abstract 2

Four new Ni-substituted Krebs-type sandwich-tungstobismuthates functionalized with amino acids have been synthesized by one-pot solution methods. By varying the number of Ni-centers and the type of organic ligands, we investigated their influence on antibacterial activity against 2 Gram-positive and 2 Gram-negative bacteria.

## Introduction

Polyoxometalates (POMs) are early transition metal oxo-complexes, which are mainly divided into two groups: isopolyoxoanions {M_*m*_O_*y*_} and heteropolyoxoanions {X_*x*_M_*m*_O_*y*_}, where X is the hetero- and M the addenda ion [mostly V, Mo, or W in their highest oxidation state (d^0^, d^1^)].^1^ Among the diversity of POM structures, the Keggin-type^2^ polyoxotungstate (POT) with the general formula [XW_12_O_40_]^*n*−^ (X = P^5+^, Si^4+^, Al^3+^, etc.; *n* = the formal charge of a molecule) and its lacunary forms have been studied thoroughly,^3^ including their monolacunary [XW_11_O_39_]^*n*−^, dilacunary [XW_10_O_36_]^*n*−^, and trilacunary [XW_9_O_34_]^*n*−^ derivatives. Lacunary structures have received extensive attention, owing to their application in retention of nanoparticles,^4^ smart inorganic surfactants,^5^ and medicinal chemistry.^6−8^ These lacunary derivatives are obtained by the removal of one or more addenda ion groups {W^VI^=O} from the plenary Keggin anion, leading to POT structures containing vacant addenda metal sites. Two trilacunary Keggin-type subunits [α/β-XW_9_O_33_]^*n*−^ (X = Bi^3+^, Si^4+^, P^5+^, etc.) can form in the presence of transition metal (TM) ions M^n+^ (e.g., Mn^2+^, Fe^3+^, Ni^2+^, and Al^3+^) four different types of sandwich-type structures: (A) Weakley-type [M_4_(B-α-XW_9_O_34_)_2_]^*n*−^,^9^ (B) Krebs-type [M_2_(WO_2_)_2_(B-β-XW_9_O_33_)_2_]^*n*−^,^10^ (C) Knoth-type [M_3_(A-α/β-XW_9_O_34_)_2_]^*n*−^,^11^ and (D) Hervé-type [M_3_(B-α-XW_9_O_33_)_2_]^*n*−^^12^ (M = Mn^2+^, Fe^3+^, Co^2+^, Ni^2+^, etc.)^13^ (Figure S1). In Krebs-type POMs, the templating heteroatom X (X = As^3+^, Sb^3+^, Bi^3+^) slides two trilacunary subunits apart from the perpendicular position, which is due to the repulsive effect of the heteroatom lone pairs. The incorporation of TM ions into the equatorial positions (two inner *cis* and two outer *fac*—Figure S1B) of Krebs-type POMs has resulted in the formation of a variety of 3d-metal-disubstituted Krebs-type POMs,^14,15^ where 3d-metals are not shielded by POM scaffold, and the number of incorporated TM-ions can be up to four. This incorporation of TM ions affects Krebs-type POMs’ properties, such as their catalytic performance,^16^ molecular magnetism properties,^17^ and activity in biological systems.^18^ Considering the previously demonstrated inhibitory activity of Ni(II)-containing Schiff base complexes^19^ and nanoparticles^20^ against Gram-positive and Gram-negative bacteria,^17,18^ applying Ni^2+^ in POM structures may endow them with potentially high antibacterial properties.

The nuclearity, shape, and size of POMs’ structures can be modified by organic components.^21,22^ Organic–inorganic POM hybrids^23^ offer a variety of options for integrating POMs into functional architectures and applications in catalysis,^24^ material science,^25^ and medicinal chemistry regarding their antibacterial^26−28^ and anticancer^29^ properties. From a drug design standpoint, POMs benefit from functionalization with organic moieties in several ways, including reduced long-term toxicity by increasing clearance for better degradation,^30^ higher activity,^26^ and selectivity toward specific biological targets.^31^

TM-substituted Krebs-type POMs’ organic functionalization based on amino acids (AA) may spark their medicinal activity. AA as N,O-chelating organic ligands can coordinate directly to the TM ions in equatorial position of Krebs-type POMs and replace labile water molecules in their coordination sphere.^32,33^ AAs are biologically active as neurotransmitters,^34^ signaling molecules,^35^ and antioxidants,^36^ making them excellent candidates as the organic functionalizing agents. While functionalizing POMs with AAs offers potential benefits, there are currently only a limited number of reported examples of AA-functionalized inorganic–organic hybrid POMs. Some reported examples include the coupling of β-alanine, l-alanine, glycine, and l-lysine to the Anderson-type POM [XMo_6_O_21_(AA)_3_]^*n*−^ (*n* = 2, X = Se^4+^, Te^4+^; *n* = 3, X = As^3+^, Sb^3+^, Bi^3+^),^37^ Fe^3+^ containing Krebs-type POMs hybridized with threonine ([Fe_4_(H_2_O)_8_(l-thr)_2_(B-β-XW_9_O_33_)_2_]^6–^; l-thr = l-threonine and X = As^3+^ or Sb^3+^),^38,39^ glycine or enantiopure l- or d-norleucine (l-Nle or d-Nle) containing giant mixed-metal POMs [As_4_(M_4_)Mo^VI^_*x*_W^VI^_44–*x*_Y_4_O_160_(AA)_*y*_(H_2_O)_*z*_]^*n*−^ (M = combination of Mo, W, and Y; AA = α-amino acid; *x* = 0–3; *y* = 8, 9);^40^dl-proline functionalized octamolybdate;^41^ and glycine functionalized decavanadate.^42^

**Figure 1:**
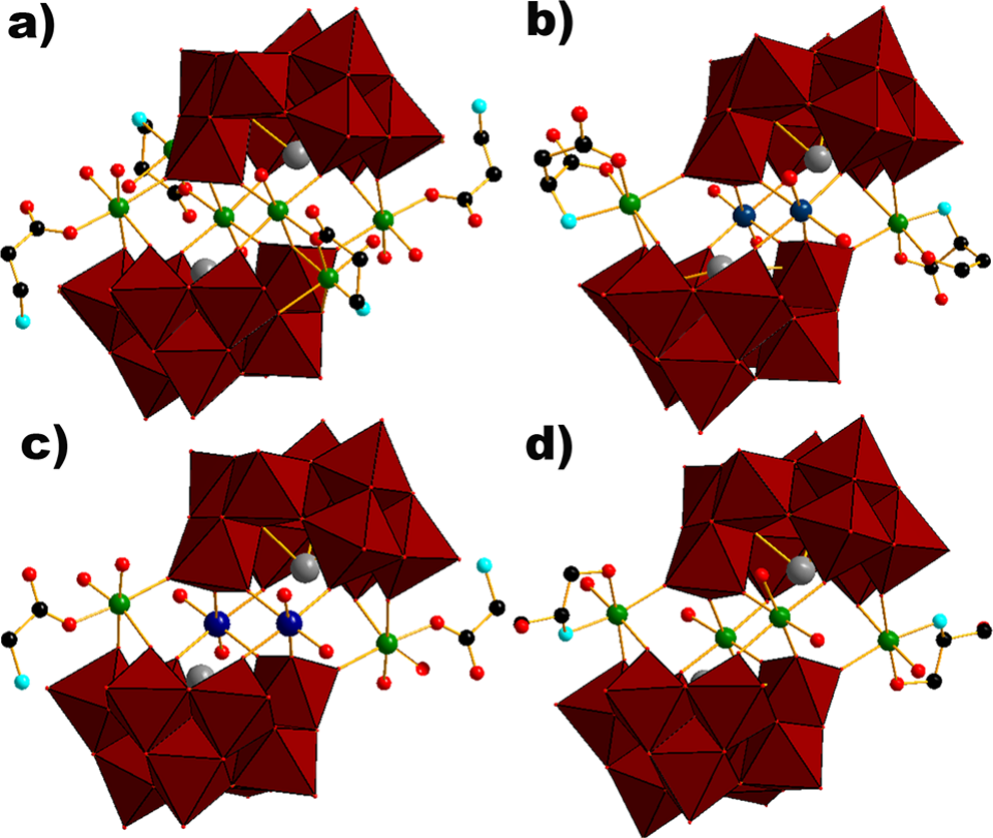
Polyhedral and ball-and-stick representation of nickel-Krebs-type TBs hybridized with: (A) β-alanine in **{(β-ala)**_**4**_**(Ni**_**3**_**)**_**2**_**(BiW**_**9**_**)**_**2**_**}**, (B) l-aspartic acid in **{(****l****-asp)**_**2**_**(NiW)**_**2**_**(BiW**_**9**_**)**_**2**_**}**, (C) glycine in **{(gly)**_**2**_**(NiW)**_**2**_**(BiW**_**9**_**)**_**2**_**}** and (D) serinol in **{(serinol)**_**2**_**Ni**_**4**_**(BiW**_**9**_**)**_**2**_**}**. Color code: red polyhedral, {WO_6_}; blue spheres, tungsten; gray spheres, bismuth; red spheres, oxygen; turquoise spheres, nitrogen; black spheres, carbon; green spheres, nickel.

This work was set to synthesize a series of hybrid Krebs-type POTs with AA coordinated to Ni^2+^ ions in the equatorial position. We report on the synthesis and characterization of four Ni-based Krebs-type tungstobismuthates (TBs) hybridized with the amino acids: β-alanine in **{(β-ala)**_**4**_**(Ni**_**3**_**)**_**2**_**(BiW**_**9**_**)**_**2**_**}**, l-aspartic acid in **{(****l****-asp)**_**2**_**(NiW)**_**2**_**(BiW**_**9**_**)**_**2**_**}**, glycine in **{(gly)**_**2**_**(NiW)**_**2**_**(BiW**_**9**_**)**_**2**_**}**, and serinol,^43^ a structural analog to the amino acid serine, in **{(serinol)**_**2**_**Ni**_**4**_**(BiW**_**9**_**)**_**2**_**}**, which are all shown in Figure 1.

## Results and Discussion

### Synthesis

Since each amino acid’s side chain affects its behavior, coordination of different amino acids to the TM ions of Krebs-type POT cannot be achieved by a single protocol; therefore, three distinct synthesis approaches were applied (Scheme S1). Using approach I, **{(β-ala)**_**4**_**(Ni**_**3**_**)**_**2**_**(BiW**_**9**_**)**_**2**_**}** was prepared by adding Ni^2+^ ions to an aqueous solution containing framework ions WO_4_^2–^, the heteroion Bi^3+^, and the ligand β-alanine in a ratio of 10:1.5:8:10 W:Bi:Ni:β-ala at pH 5 (Scheme S1A). Since the compounds **{(****l****-asp)**_**2**_**(NiW)**_**2**_**(BiW**_**9**_**)**_**2**_**}**, **{(gly)**_**2**_**(NiW)**_**2**_**(BiW**_**9**_**)**_**2**_**}**, and **{(serinol)**_**2**_**Ni**_**4**_**(BiW**_**9**_**)**_**2**_**}** could not be crystallized with the first method, a modified approach was chosen. In the second approach, **{(****l****-asp)**_**2**_**(NiW)**_**2**_**(BiW**_**9**_**)**_**2**_**}** was synthesized by reacting the starting materials in a ratio of 10:1:3.3:10 W:Bi:Ni:L-asp; however, the framework ions WO_4_^2–^ and the heteroion Bi^3+^ were let to react with one another first. Later, the solution was adjusted to pH 6.5 along with the addition of Ni^2+^ ions and the ligand l-aspartic acid, and subsequently, the pH was reduced to 5 (Scheme S1B). **{(gly)**_**2**_**(NiW)**_**2**_**(BiW**_**9**_**)**_**2**_**}** and **{(serinol)**_**2**_**Ni**_**4**_**(BiW**_**9**_**)**_**2**_**}** were synthesized by adding Ni^2+^ ions to an sodium acetate−acetic acid buffer solution (1 M NaOAc/HOAc, pH 5.5) containing WO_4_^2–^, Bi^3+^, and the ligand (glycine or serinol) in a ratio of 10:1.5:8:10 W:Bi:Ni:L (approach III) (Scheme S1C). The formation of all products was highly dependent on the respective pH of the solution, where minor pH changes (0.5 unit) hampered the coordination of AA to Ni^2+^ and resulted in unfunctionalized Krebs-type TB.

### X-Ray Crystallography

**Figure 2:**
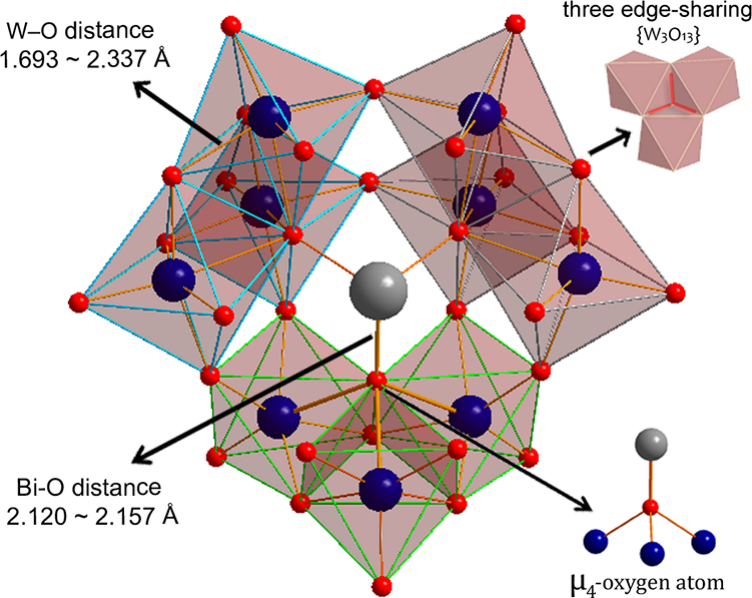
Depiction of three edge-sharing {W_3_O_13_} groups, W–O distance, as well as Bi–O distance in respect of the three {W_3_O_13_} groups in each trilacunary unit. Color code: red transparent polyhedral, {WO_6_} (different colors for polyhedrals’ borderlines are applied to isolate each {W_3_O_13_} group); blue spheres, tungsten; red spheres, oxygen; gray sphere, bismuth.

Single-crystal X-ray diffraction (SXRD) studies were performed (Table S3) on **{(β-ala)_4_(Ni_3_)_2_(BiW_9_)_2_}** (Tables S4, S5), **{(l-asp)_2_(NiW)_2_(BiW_9_)_2_}** (Tables S6, S7), **{(gly)_2_(NiW)_2_(BiW_9_)_2_}** (Tables S8, S9), and **{(serinol)_2_Ni_4_(BiW_9_)_2_}** (Tables S10, S11). SXRD reveals that **{(β-ala)_4_(Ni_3_)_2_(BiW_9_)_2_}** and **{(serinol)_2_Ni_4_(BiW_9_)_2_}** crystallize in the triclinic space group *P*1̅, while **{(l-asp)_2_(NiW)_2_(BiW_9_)_2_}** and **{(gly)_2_(NiW)_2_(BiW_9_)_2_}** belong to the monoclinic space groups *P*2_1_ and *C*2/*m*, respectively. In all four POTs, each Krebs-type construction comprises two trilacunary moieties {*B*-β-BiW_9_O_33_} consisting of three edge-sharing {W_3_O_13_} groups surrounding a Bi^3+^ in the center and resulting in an overall *C*_2*v*_ symmetry of the anion (Figure 2). All W^6+^ centers exhibit an octahedral coordination environment with W–O distances ranging from 1.693 to 2.337 Å. Bi^3+^ is bonding three μ_4_-oxygen ions from three {W_3_O_13_} groups with Bi–O distances ranging from 2.120 to 2.157 Å (Figure 2). Four Ni^2+^ ions (equatorial position) in **{(β-ala)_4_(Ni_3_)_2_(BiW_9_)_2_}** (Figure 1a) and **{(serinol)_2_Ni_4_(BiW_9_)_2_}** (Figure 1d) link two trilacunary moieties, whereas two inner W^6+^ ions (*cis* position) and two peripheral Ni^2+^ ions (*fac* position) connect two trilacunary moieties in **{(l-asp)_2_(NiW)_2_(BiW_9_)_2_}** (Figure 1b) and **{(gly)_2_(NiW)_2_(BiW_9_)_2_}** (Figure 1c). In all four compounds, all Ni^2+^ ions exhibit an octahedral coordination environment.

**Figure 3:**
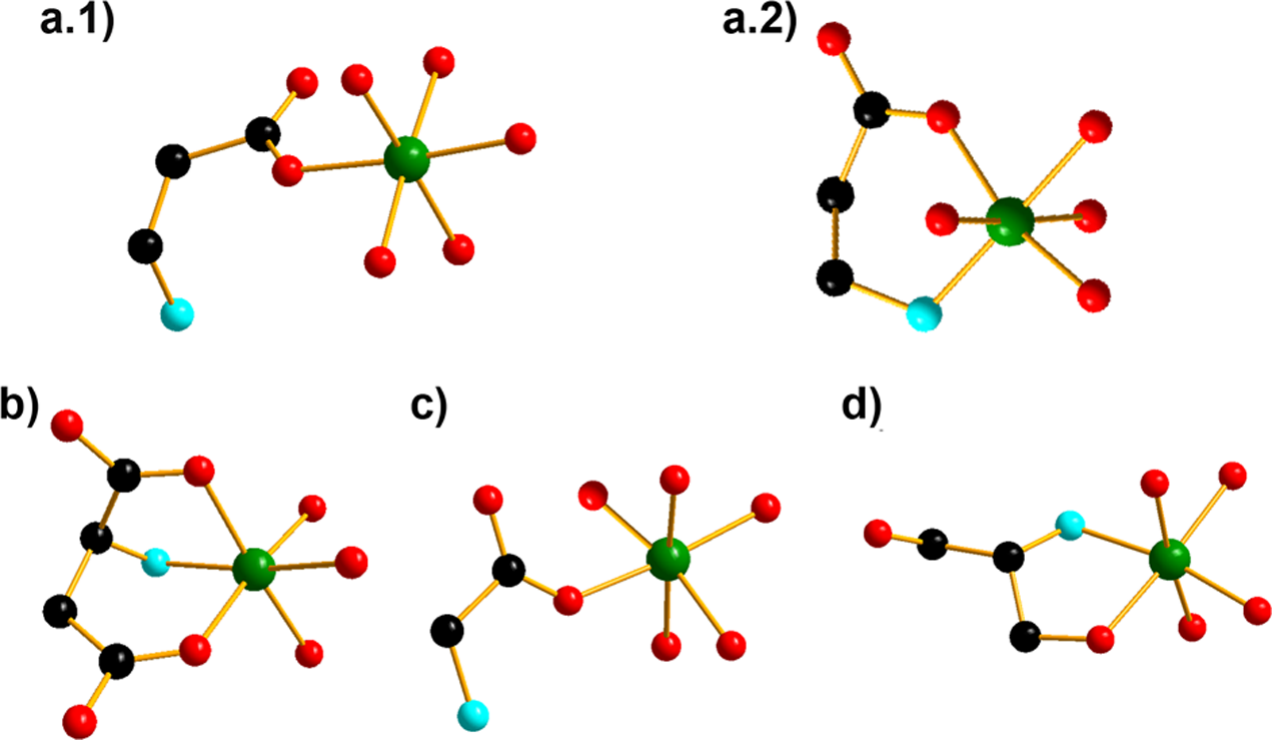
Ball-and-stick representation of coordination environment around nickel in all compounds. (a.1) Monodentate and (a.2) bidentate coordination of β-alanine to the nickel ion in equatorial and lateral position in **{(β-ala)**_**4**_**(Ni**_**3**_**)**_**2**_**(BiW**_**9**_**)**_**2**_**}**, respectively. (b) Coordination of l-aspartic acid as tridentate ligand to nickel in **{(L-asp)**_**2**_**(NiW)**_**2**_**(BiW**_**9**_**)**_**2**_**}**. (c) Coordination of glycine as monodentate ligand to the nickel ion in **{(gly)**_**2**_**(NiW)**_**2**_**(BiW**_**9**_**)**_**2**_**}**. (d) Coordination of serinol as bidentate ligand to nickel ion in **{(serinol)**_**2**_**Ni**_**4**_**(BiW**_**9**_**)**_**2**_**}**. Color code: red spheres, oxygen; black spheres, carbon; turquoise spheres, nitrogen; green spheres, nickel.

The AAs show different modes of chelation in each compound (Figure 3). In **{(β-ala)**_**4**_**(Ni**_**3**_**)**_**2**_**(BiW**_**9**_**)**_**2**_**}**, β-alanine is coordinated in two distinct modes to the nickel ions. In equatorial position, β-alanine is bound to Ni^2+^ monodentally via the carboxyl group exhibiting a distance of 2.061 Å (Figure 3a.1). In lateral position, β-alanine is bound to Ni^2+^ bidentally via amino-N and carboxylate-O atoms exhibiting a distance of 2.047 and 1.999 Å, respectively (Figure 3a.2). In compound **{(****l****-asp)**_**2**_**(NiW)**_**2**_**(BiW**_**9**_**)**_**2**_**},**l-aspartic acid is coordinated to Ni^2+^ as a tridentate ligand via the α-amino-N, the α-carboxylate-O atoms, and the β-carboxylate-O atom from the side chain with the distances of 2.076, 2.062, and 2.040 Å, respectively (Figure 3b). In **{(gly)**_**2**_**(NiW)**_**2**_**(BiW**_**9**_**)**_**2**_**},** glycine hybridized the POT anion by coordinating with the Ni^2+^ as a monodentate ligand via a carboxylate-O donor atom with a distance of 1.990 Å (Figure 3c). In **{(serinol)**_**2**_**Ni**_**4**_**(BiW**_**9**_**)**_**2**_**},** serinol is attached to Ni^2+^ as a bidentate ligand via nitrogen and oxygen with the distances of 2.070 and 2.072 Å, respectively (Figure 3d). All Ni–O/N bond distances are listed in Table S12.

**Figure 4:**
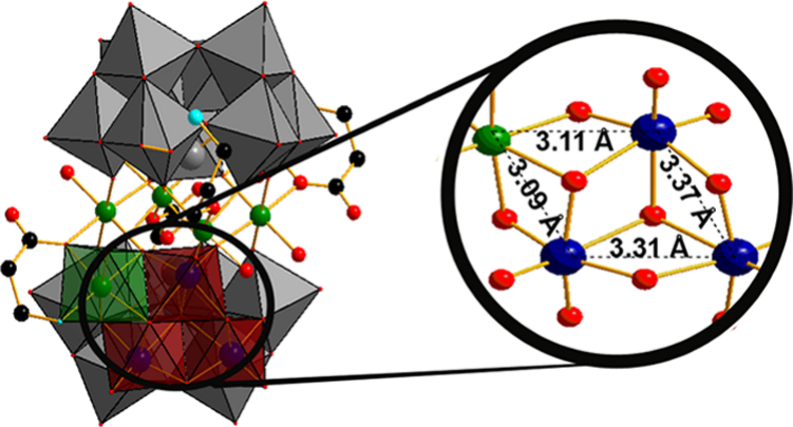
Representation of octahedral coordinated nickel bonded edge-shared to the {W_3_O_13_} unit. Color code: red and gray polyhedra, {WO_6_} (this contrast is used to clarify the position of lateral nickel in respect of the other {W_3_O_13_} group); green polyhedra, {NiO_5_N}; gray spheres, bismuth; blue spheres, tungsten; red spheres, oxygen; turquoise sphere, nitrogen; green spheres, nickel.

Compound **{(β-ala)**_**4**_**(Ni**_**3**_**)**_**2**_**(BiW**_**9**_**)**_**2**_**}** exhibits a unique structure. According to the structure analyses, **{(β-ala)**_**4**_**(Ni**_**3**_**)**_**2**_**(BiW**_**9**_**)**_**2**_**}** indicates a distinctly new pattern as a Krebs-type POT compared to pure inorganic Krebs-type POT by settling four nickel ions in equatorial position between two trivacant Keggin subunits {*B*-β-BiW_9_O_33_} along with two nickel ions in lateral position. Both laterally coordinated Ni^2+^ ions are located next to the {W_3_O_13_} groups in each trilacunary Keggin subunit to form a rhombohedral core (Figure 4). To the best of our knowledge, Krebs-type POMs with six inserted TM ions have not been reported to date.

Powder XRD measurements were performed on all compounds and their comparison with the corresponding simulated sample shows high compatibility for both compounds **{(****l****-asp)**_**2**_**(NiW)**_**2**_**(BiW**_**9**_**)**_**2**_**}** and **{(gly)**_**2**_**(NiW)**_**2**_**(BiW**_**9**_**)**_**2**_**}** (Figures S10, S11), while the diffractograms of **{(β-ala)**_**4**_**(Ni**_**3**_**)**_**2**_**(BiW**_**9**_**)**_**2**_**}** and **{(serinol)**_**2**_**Ni**_**4**_**(BiW**_**9**_**)**_**2**_**}** do not match the theoretical values (Figures S9, S12), possibly due to the factors such as scanning speed, preferred orientation, crystal blooming, and loss of water molecules leading to crystal lattice dissolution.

### Infrared Spectroscopy

All four Ni-substituted Krebs-type TB display the characteristic absorption bands in the fingerprint region between 1000 and 400 cm^–1^, which correspond to W–O–W and W=O stretching vibration.^44^ The strong broad bands between 3600 and 2500 cm^–1^ and the sharp peak at about 1600 cm^–1^ are attributed to O–H stretching and H–O–H bending vibration of lattice water in all four POMs. A series of weak and medium bands ranging from 1270 to 1500 cm^–1^ are assigned to the C–O, C–N stretching vibration and H–C–H bending vibration in amino acids^45^ (Figures S2–S5, Table S1).

### Thermogravimetric Analysis

The thermogravimetric analyses (TGAs) have been studied in the temperature range from 25 to 700 °C (Table S2). In the thermogram of **{(β-ala)**_**4**_**(Ni**_**3**_**)**_**2**_**(BiW**_**9**_**)**_**2**_**},** the mass loss of 9.43% in the range of 34–97 °C is associated with the release of 35.5 lattice water molecules. The mass loss of 10.93% in the range of 98–691 °C is assigned to the removal of other 13.5 lattice water molecules, 8 coordination water molecules, and the decomposition of four β-alanine ligands (Figure S6). In **{(****l****-asp)**_**2**_**(NiW)**_**2**_**(BiW**_**9**_**)**_**2**_**}**, a large loss of 6.11% in the temperature range of 25–111 °C is attributed to the removal of 23 lattice water molecules. The weight loss of 8.8% in the range of 112–630 °C is related to the removal of 10 existing lattice water molecules and the decomposition of 3 l-aspartic acid ligands (Figure S7). Compound **{(gly)**_**2**_**(NiW)**_**2**_**(BiW**_**9**_**)**_**2**_**}** lost 8.17% of its mass at the temperature range of 23–112 °C, which is assigned to the loss of 33.5 lattice water molecules. Subsequently, in the temperature range of 113–421 °C, it loses 15.81% of its mass due to the release of 41.5 lattice water molecules, four bound water molecules, and the removal of two glycine ligands (Figure S8). In **{(serinol)**_**2**_**Ni**_**4**_**(BiW**_**9**_**)**_**2**_**}**, the weight loss of 11.9% in the range from 25 to 312 °C is associated with 42 lattice water molecules. The weight loss of 4.4% in the range of 313–528 °C is assigned to the removal of six coordinated water molecules and the decomposition of two serinol ligands (Figure S9).

### Vis Spectroscopy

The vis spectrum of 3 mM aqueous solution of **{(****l****-asp)**_**2**_**(NiW)**_**2**_**(BiW**_**9**_**)**_**2**_**}** shows the absorption band from 550 to 850 nm with the maximum intensity 0.045, which is related to charge transfer oxygen → Ni^2+^^46^ transitions. The absorbance was tracked for 18 h to investigate the compound’s stability in an aqueous solution. After 18 h, maximum absorption is increased to 0.076 and one shoulder band appears with a maximum absorbance of 0.075 in the range from 715 to 790 nm (Figure S13). The appearance of a shoulder peak and concurrent increase in absorbance could be attributed to the release of nickel ions from Krebs-type anion and its partial hydration, which is fairly close to the vis spectrum of nickel nitrate aqueous solution (6 mM) (Figure S14). The observed possible detachment of AA in **{(****l****-asp)**_**2**_**(NiW)**_**2**_**(BiW**_**9**_**)**_**2**_**}** is consistent with the reported low stability of transition metal complexes that form carboxylate bonds in solution.^47^ Due to solubility issues (max. conc. before solution became cloudy = 0.75 mM), this absorption has not been observed in compounds **{(β-ala)**_**4**_**(Ni**_**3**_**)**_**2**_**(BiW**_**9**_**)**_**2**_**}**, **{(gly)**_**2**_**(NiW)**_**2**_**(BiW**_**9**_**)**_**2**_**},** and **{(serinol)**_**2**_**Ni**_**4**_**(BiW**_**9**_**)**_**2**_**}** (Figure S15). The stability of **(****l****-asp)**_**2**_**(NiW)**_**2**_**(BiW**_**9**_**)**_**2**_ in Mueller–Hinton Broth medium (MHB)^48^ for determining minimal inhibitory concentrations (at 37 °C—18 h) was studied as well. The maximum absorption increased from 0.042 to 0.075 under the applied conditions; the increase in absorption may be caused by the dissociation of l-aspartic acid from the Krebs-type anion or a change in its chelation mode toward the nickel ion, which changes the nickel ion coordination environment and absorption (Figure S16). While **(****l****-asp)**_**2**_**(NiW)**_**2**_**(BiW**_**9**_**)**_**2**_ exhibits instability in both water and MHB, we have observed that the compound remains stable for at least 18 h at 25 °C in HEPES buffer at pH 7.4 (Figures S17 and S18). Therefore, we recommend the use of HEPES buffer for biological testing of this compound due to its increased stability under these conditions.

### Antibacterial Testing

Antibacterial tests were done on two Gram-positive bacteria—*Staphylococcus aureus*, which causes a wide range of clinical manifestations,^49^ and *Enterococcus faecalis*, which leads to life-threatening infections, particularly in the nosocomial environment.^50^ In addition, to these, tests were also done on two strains of the Gram-negative bacteria—*Moraxella catarrhalis*, which induces respiratory tract infection,^51^ and *Escherichia coli*, often linked to several epidemic diseases involving foodborne illness and diarrhea.^52^ The antibacterial activity of compounds **{(β-ala)**_**4**_**(Ni**_**3**_**)**_**2**_**(BiW**_**9**_**)**_**2**_**}**, **{(****l****-asp)**_**2**_**(NiW)**_**2**_**(BiW**_**9**_**)**_**2**_**}**, **{(gly)**_**2**_**(NiW)**_**2**_**(BiW**_**9**_**)**_**2**_**}**, and **{(serinol)**_**2**_**Ni**_**4**_**(BiW**_**9**_**)**_**2**_**}** was tested and compared with the activity of unfunctionalized Ni-substituted Krebs-type POT Na_6_H_4_[{Ni(H_2_O)_3_}_2_(WO_2_)_2_(*B*-β-BiW_9_O_33_)_2_]·36H_2_O^53^**{(NiW)**_**2**_**(BiW**_**9**_**)**_**2**_**}**, as well as with all inorganic salt precursors and organic ligands to study the influence of each individual component (Tables 1, S13). The antibacterial activity of unfunctionalized **{(NiW)**_**2**_**(BiW**_**9**_**)**_**2**_**}** (MiCs are 256 and 128 μg/mL) compared to di- **{(****l****-asp)**_**2**_**(NiW)**_**2**_**(BiW**_**9**_**)**_**2**_**}** (MIC > 1024 μg/mL), **{(gly)**_**2**_**(NiW)**_**2**_**(BiW**_**9**_**)**_**2**_**}** (MIC > 1024 μg/mL), and tetra-substituted Ni-sandwiches **{(serinol)**_**2**_**Ni**_**4**_**(BiW**_**9**_**)**_**2**_**}** (MIC > 1024 μg/mL) (Tables 1, S13) is slightly higher against both tested Gram-positive bacteria, but shows no significant superiority against tested Gram-negative bacteria. The observed lower antibacterial response for **{(L-asp)**_**2**_**(NiW)**_**2**_**(BiW**_**9**_**)**_**2**_**}**, **{(gly)**_**2**_**(NiW)**_**2**_**(BiW**_**9**_**)**_**2**_**}**, and **{(serinol)**_**2**_**Ni**_**4**_**(BiW**_**9**_**)**_**2**_**}** could be due to the shielding of nickel ions by AA ligands in Krebs-type POT and their lower solubility in H_2_O (Table S13). The higher antibacterial activity of **{(β-ala)**_**4**_**(Ni**_**3**_**)**_**2**_**(BiW**_**9**_**)**_**2**_**}** (MICs are in the range 8–256 μg/mL) compared to **{(****l****-asp)**_**2**_**(NiW)**_**2**_**(BiW**_**9**_**)**_**2**_**}**, **{(gly)**_**2**_**(NiW)**_**2**_**(BiW**_**9**_**)**_**2**_**}**, **{(serinol)**_**2**_**Ni**_**4**_**(BiW**_**9**_**)**_**2**_**},** and **{(NiW)**_**2**_**(BiW**_**9**_**)**_**2**_**}** could be attributed to the higher number of nickel centers in its structure.^54^

**Table 1:** Minimum Inhibitory Concentrations (MICs) of {(β-ala)_4_(Ni_3_)_2_(BiW_9_)_2_}, {(l-asp)_2_(NiW)_2_(BiW_9_)_2_}, {(gly)_2_(NiW)_2_(BiW_9_)_2_}, {(serinol)_2_Ni_4_(BiW_9_)_2_}, {(NiW)_2_(BiW_9_)_2_}^53^ against *Staphylococcus aureus* (*S. aureus*-ATCC 13709), *Enterococcus faecalis* (*E. faecalis*-ATCC 29212), *Moraxella catarrhalis* (*M. catarrhalis*-ATCC 23246), and *Escherichia coli* (*E. coli*-TolC-Tn10)

	MIC (μg/mL)			
compound	*S. aureus*-ATCC 13709	*E. faecalis*-ATCC 29212	*M. catarrhalis*-ATCC 23246	*E. coli* TolC-Tn10
**{(β-ala)_4_(Ni_3_)_2_(BiW_9_)_2_}**	256	32	8	>256
**{(l-asp)_2_(NiW)_2_(BiW_9_)_2_}**	>1024	>1024	128	>1024
**{(gly)_2_(NiW)_2_(BiW_9_)_2_}**	>1024	>1024	256	>1024
**{(serinol)_2_Ni_4_(BiW_9_)_2_}**	>1024	>1024	512	>1024
**{(NiW)_2_(BiW_9_)_2_}**	256	128	128	>1024

## Conclusions

In conclusion, a series of functionalized POTs with AA was synthesized and characterized. We demonstrated for the first time that amino acids can be covalently attached to nickel in Krebs-type TBs. The MIC values show that the antibacterial activity of compounds **{(****l****-asp)**_**2**_**(NiW)**_**2**_**(BiW**_**9**_**)**_**2**_**}**, **{(gly)**_**2**_**(NiW)**_**2**_**(BiW**_**9**_**)**_**2**_**}**, and **{(serinol)**_**2**_**Ni**_**4**_**(BiW**_**9**_**)**_**2**_**}** has decreased compared to unfunctionalized parent Ni-Krebs anion, which could be due to the shielding of nickel centers by ligands, whereas **{(β-ala)**_**4**_**(Ni**_**3**_**)**_**2**_**(BiW**_**9**_**)**_**2**_**}** exhibits higher activity, which may be due to the presence of six nickel ions in its structure. Because of their unique structures containing biologically relevant ligands (AA) and highly negative charge, the herein-reported POT clusters might be capable of interacting with proteins. As a result, they may theoretically stabilize new protein regions previously inaccessible to POMs, potentially resulting in the formation of new POT-protein crystals.
