# Supplementary material for: Synthesis, Characterization, and Antibacterial Activity of Ni-Substituted Krebs-type Sandwich-Tungstobismuthates Functionalized with Amino Acids
Source: Inorg Chem. 2023 Jun 7;62(24):9484–90. doi: 10.1021/acs.inorgchem.3c00747 (PMC10283016; doi:10.1021/acs.inorgchem.3c00747)
Supplement: Supplementary file 1 — ic3c00747_si_001.pdf [file ic3c00747_si_001.pdf]

Synthesis, Characterization, and Antibacterial Activity of Ni-Substituted Krebs-Type Sandwich-Tungstobismuthates Functionalized With Amino Acids

Morteza Rafieeshad[1], Nadiia I. Gumerova[1], Elias Tanuhadi[1], Gerald Giester[2], Hana Čipčić-Paljetak[3], Donatella Verbanac[4] and Annette Rompel[1]*

[1]Universität Wien, Fakultät für Chemie, Institut für Biophysikalische Chemie, 1090 Wien, Austria. www.bpc.univie.ac.at, Email: annette.rompel@univie.ac.at

[2]Universität Wien, Fakultät für Geowissenschaften, Geographie und Astronomie, Institut für Mineralogie und Kristallographie, 1090 Wien, Austria.

[3]Center for Translational and Clinical Research, University of Zagreb School of Medicine, Šalata 2, 10000 Zagreb, Croatia.

[4] University of Zagreb Faculty of Pharmacy and Biochemistry, A. Kovačića 1, 10000 Zagreb, Croatia

*Correspondence e-mail: annette.rompel@univie.ac.at

Content

1. General information 3

2. Experimental Procedure 6

2.1 Preparation of $K_4Ni_2[\{Ni(\beta\text{-ala})(H_2O)_2\}_2\{Ni(H_2O)\}_2\{Ni(H_2O)(\eta^2\text{-}\beta\text{-ala})\}_2(B\text{-}\beta\text{-}BiW_9O_{33})_2]\cdot 49H_2O$ **$\{(\beta\text{-ala})_4(Ni_3)_2(BiW_9)_2\}$** 6

2.2 Preparation of $K_{3.5}Na_{6.5}[\{Ni(\eta^3\text{-L-asp})\}_2(WO_2)_2(B\text{-}\beta\text{-}BiW_9O_{33})_2]\cdot 36H_2O\cdot$L-asp **$\{(L\text{-asp})_2(NiW)_2(BiW_9)_2\}$** 6

2.3 Preparation of $K_4Na_6[\{Ni(gly)(H_2O)_2\}_2(WO_2)_2(B\text{-}\beta\text{-}BiW_9O_{33})_2]\cdot 86H_2O$ **$\{(gly)_2(NiW)_2(BiW_9)_2\}$** 6

2.4 Preparation of $K_2Na_8[\{Ni(\eta^2\text{-serinol})(H_2O)\}_2\{Ni(H_2O)_2\}_2(B\text{-}\beta\text{-}BiW_9O_{33})_2]\cdot 42H_2O$ **$\{(serinol)_2Ni_4(BiW_9)_2\}$** 7

3. IR Spectra 8

4. Thermogravimetric analysis 11

5. Single-Crystal X-ray Diffraction 16

6. Powder X-ray Diffraction 21

7. Vis Spectroscopy and ^{1}H NMR spectroscopy 25

8. Antibacterial activity 31

9. References 32

1. General information

All reagents were obtained commercially from Sigma Aldrich (Austria), AlfaAesar and Merck with a high-purity grade and were used as purchased without further purification. Unfunctionalized Ni-Krebs POT $Na_6H_4[\{Ni(H_2O)_3\}_2(WO_2)_2(B\text{-}\beta\text{-}BiW_9O_{33})_2]\cdot 36H_2O$ **$\{(NiW)_2(BiW_9)_2\}$** has been synthesized according to the published procedure.[1]

Elemental analysis (EA): Elemental analysis was performed with an iCAP 6500 series inductively coupled plasma-optical emission spectrometry (ICP-OES) spectrometer (Thermo Scientific, USA). The ICPOES was equipped with a standard sample introduction system consisting of a concentric nebulizer and a cyclonic spray chamber. Transportation of sample solutions was performed by the peristaltic pump of the iCAP 6500 coupled to an ASX-520 auto sampler (Cetac, USA). Per element two sensitive and non-interfered emission lines were used, the first line for measurement and the second line for quality control. Elemental microanalysis of C/H/N/O contents was performed by Mikroanalytisches Laboratorium (University of Vienna, Faculty of Chemistry). An EA 3000 (Eurovector) was used for C/H/N/S-analysis. O-determination was performed by high-temperature digestion using the HT 1500 (Hekatech, Germany) pyrolysis system in combination with the EA 3000 system.

Attenuated total reflection Fourier-transform Infrared Spectroscopy (ATR-IR): All ATR-IR spectra were recorded on a Bruker Vertex70 IR Spectrometer equipped with a single-reflection diamond-ATR unit. Frequencies are given in cm^{-1}, intensities denoted as w = weak, m = medium, s = strong.

Thermogravimetric analysis (TGA): was performed on a Mettler SDTA851e Thermogravimetric Analyzer under air and N_2 with a heating rate of 5 °C min^{-1} in the region 25-700 °C. These results were then used to calculate the number of crystal waters present in each compound.

Single crystal X-ray diffraction (SXRD): X-ray intensity data were measured on a Bruker APEXII diffractometer equipped with a CCD (charge-coupled device) area detector, Incoatec Microfocus Source IS (30 W, multilayer mirror, Mo-K), and an Oxford Cryosystem (Cryostream 800 Plus LT) device. The following software was used: the Bruker Apex3 suite[2] the SHELX programme suite for structure solution (SHELXT[3]), structure refinement (SHELXL[3]) and OLEX2[4] as graphical user-interface.

Powder X-ray diffraction (PXRD): PXRD was performed on a Bruker D8 ADVANCE diffractometer, Cu Kα radiation, $\lambda = 1.54056$ Å, LYNXEYE silicon strip detector and a SolX energy dispersive detector with a variable slit aperture of 12 mm, $8° \leq 2\theta \leq 50°$. The simulated pattern was obtained from the SXRD data collected on single-crystal of each compound using Mercury programme[5].

UV/Vis spectroscopy: UV/Vis spectra were collected on a Shimadzu UV 1800 spectrophotometer.

Minimum inhibitory concentrations (MICs) were determined by the broth microdilution method according to the guidelines of the Clinical Laboratory Standards Institute[6]. Stock solutions of compounds were prepared in 20 mg/mL or 10 mg/mL concentration in sterile H_2O. Double dilutions

of tested compounds in 96-well microtiter plates were prepared in 1024 – 2µg/ml concentration range for high molecular weight complexes. *E.coli* and *S. aureus* were grown on Mueller-Hinton agar plates (by Becton Dickinson, USA) and *E. faecalis* and *M.catarrhalis* where grown on Mueller-Hinton agar with 5% defibrinated sheep blood. Inocula were prepared by direct colony suspension method and plates were inoculated with bacteria in a final concentration of 5x10^5 CFU/mL. Results were determined by visual inspection after 20-22h incubation at 37°C in ambient air. Testing was performed by the standard broth microdilution method with azithromycin as the reference antibiotic[7] to assess test validity.

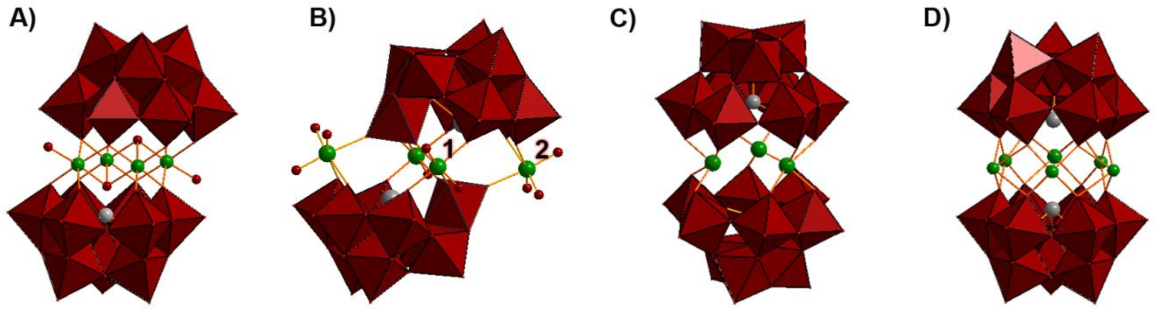

Figure S1. Polyhedral and ball-and-stick representation of four types of Keggin-based sandwich-type POMs. **A**) Weakley-type[8] $[M_4(B\text{-}\alpha\text{-}XW_9O_{34})_2]^{n-}\alpha$ (X= P(V), As(V), Si(IV), Ge(IV)); **B**) Krebs-type[9] $[M_4(B\text{-}\beta\text{-}XW_9O_{33})_2]^{n-}$, (X= Bi(III), Sb(III), As(III)), (1 and 2: two inner cis and two peripheral facial position respectively); **C**) Knoth-type[10] $[M_3(A\text{-}\alpha/\beta\text{-}XW_9O_{34})_2]^{n-}$, (X = P(V), Si(IV); **D**) Herve-type[11] $[M_3(B\text{-}\alpha\text{-}XW_9O_{33})_2]^{n-}$ (X = As(III), Sb(III), Bi(III), Se(III), Te(III)), (n = the formal charge of a molecule). Color code: red polyhedra, $\{WO_6\}$; gray spheres, heteroion X; green spheres, transition metals ions M; red spheres, oxygen.

Scheme S1. Schematic representation of three distinct synthesis approaches. **A**) The synthesis procedure 1 yielded **$\{(\beta\text{-ala})_4(Ni_3)_2(BiW_9)_2\}$**. **B**) The synthesis procedure 2 yielded **$\{(L\text{-}asp)_2(NiW)_2(BiW_9)_2\}$**. **C**) The synthesis procedure 3 yielded **$\{(gly)_2(NiW)_2(BiW_9)_2\}$** and **$\{(serinol)_2Ni_4(BiW_9)_2\}$**. Color code: red polyhedra, $\{WO_6\}$; gray spheres, bismuth; blue spheres, tungsten; green spheres, nickel; red spheres, oxygen; black spheres, carbon; turquoise sphere, nitrogen.

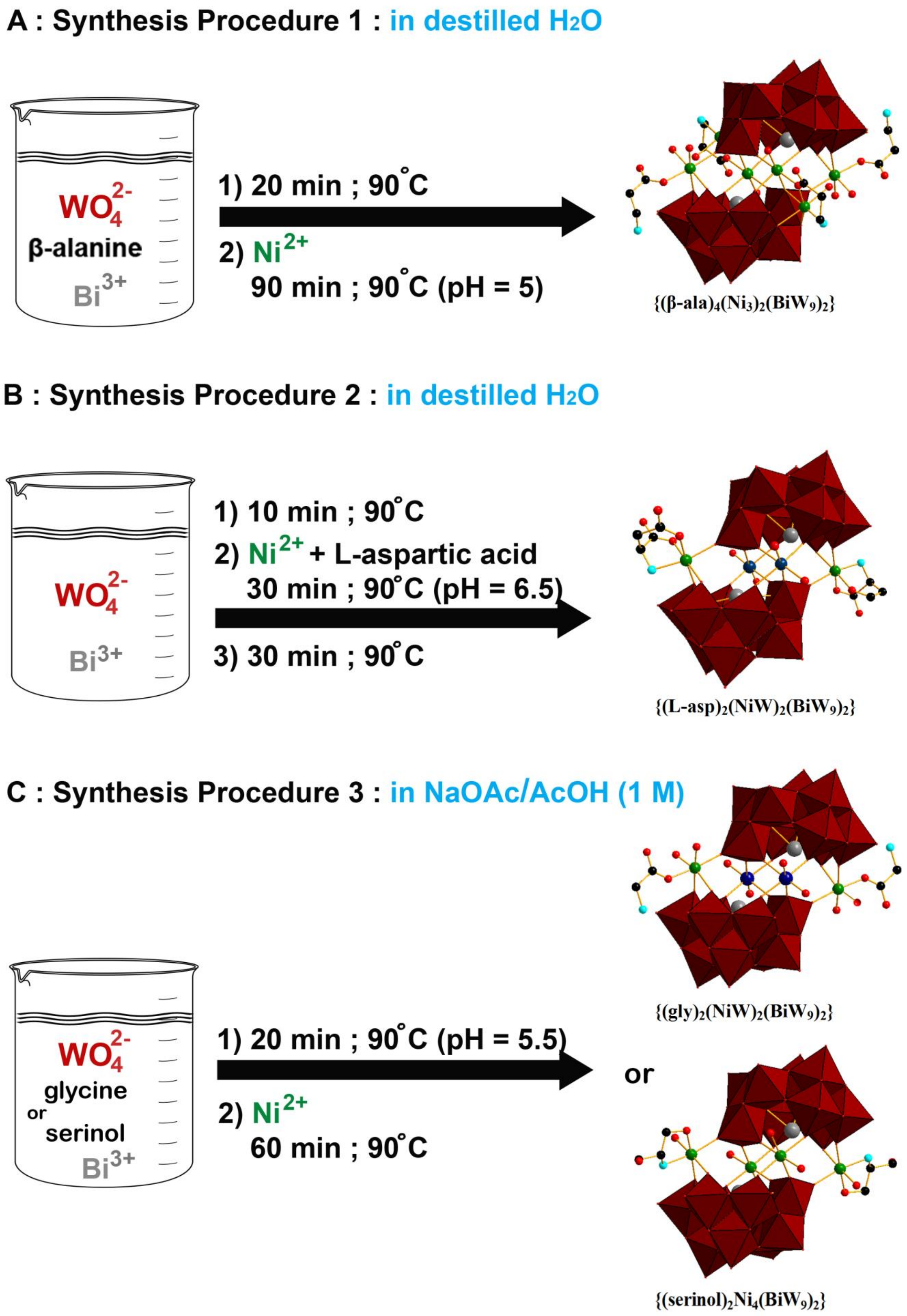
A : Synthesis Procedure 1 : in destilled H2O
WO42-
β-alanine
Bi3+
1) 20 min ; 90°C
2) Ni2+
90 min ; 90°C (pH = 5)
{(β-ala)4(Ni3)2(BiW9)2}
B : Synthesis Procedure 2 : in destilled H2O
WO42-
Bi3+
1) 10 min ; 90°C
2) Ni2+ + L-aspartic acid
30 min ; 90°C (pH = 6.5)
3) 30 min ; 90°C
{(L-asp)2(NiW)2(BiW9)2}
C : Synthesis Procedure 3 : in NaOAc/AcOH (1 M)
WO42-
glycine
or
serinol
Bi3+
1) 20 min ; 90°C (pH = 5.5)
2) Ni2+
60 min ; 90°C
or
{(gly)2(NiW)2(BiW9)2}
{(serinol)2Ni4(BiW9)2}

2. Experimental Procedure

2.1 Preparation of $K_4Ni_2[\{Ni(\beta\text{-ala})(H_2O)_2\}_2\{Ni(H_2O)\}_2\{Ni(H_2O)(\eta^2\text{-}\beta\text{-ala})\}_2(B\text{-}\beta\text{-}BiW_9O_{33})_2]\cdot 49H_2O$ $\{(\beta\text{-ala})_4(Ni_3)_2(BiW_9)_2\}$

A sample of $Na_2WO_4\cdot 2H_2O$ (0.66 g, 2 mmol) was dissolved in 20 ml distilled water. $Bi(NO_3)_3\cdot 5H_2O$ (0.14 g, 0.3 mmol), β-alanine (0.17 g, 2 mmol) and KCl (0.15 g, 2 mmol) were added to the sodium tungstate solution. The reaction mixture was heated to 90°C and stirred for 20 min. Afterwards, $Ni(NO_3)_2\cdot 6H_2O$ (0.49 g, 1.6 mmol) was added to the solution. The pH of the resulting solution was adjusted to 5 using a 1 M HCl solution. The reaction mixture was heated to 90°C again and stirred for another 60 min, then cooled to room temperature, filtered, and kept at room temperature for crystallization (**Scheme S1A**). After several days light green crystals of $K_4Ni_2[\{Ni(\beta\text{-ala})(H_2O)_2\}_2\{Ni(H_2O)\}_2\{Ni(H_2O)(\eta^2\text{-}\beta\text{-ala})\}_2(B\text{-}\beta\text{-}BiW_9O_{33})_2]\cdot 49H_2O$ were collected. Yield: 151 mg (20 % based on W). Elemental analysis calc (%) for $K_4W_{18}Bi_2Ni_8C_{12}N_4O_{131}H_{136}$ (6786 g mol^{-1}): K 2.3, W 49.7, Bi 6.2, Ni 7.0, C 2.1, N 0.8, O 31.5, H 2.0; found: K 2.5, W 50.6, Bi 6.3, Ni 7.2, C 1.3, N 0.6, O 27.4, H 1.5.

2.2 Preparation of $K_{3.5}Na_{6.5}[\{Ni(\eta^3\text{-L-asp})\}_2(WO_2)_2(B\text{-}\beta\text{-}BiW_9O_{33})_2]\cdot 36H_2O\cdot$L-asp $\{(\text{L-asp})_2(NiW)_2(BiW_9)_2\}$

Solution A: A sample of $Na_2WO_4\cdot 2H_2O$ (0.66 g, 2 mmol) was dissolved in 25 ml of distilled water and a solution of $BiONO_3$ (0.08 g, 0.2 mmol) in 2 ml of concentrated HNO_3 (65%) was added dropwise. The pH of the resulting solution was adjusted to 5 using a 1 M HCl solution. The colloid white mixture was stirred for 10 min at 90°C. Solution B: L-aspartic acid (0.26 g, 2 mmol), $Ni(NO_3)_2\cdot 6H_2O$ (0.19 g, 0.6 mmol), and KCl (0.15 g, 2 mmol) were dissolved in 10 ml of distilled water. Then solution B was added to solution A dropwise and the pH of the mixture was adjusted to 6.5 by using 1 M NaOH. The reaction mixture was heated to 90°C and stirred for 30 min. After 30 min the pH of the mixture was adjusted to 5 by using 1 M HCl and then heated again to 90°C and stirred for another 30 min, cooled to room temperature, filtered, and kept at room temperature for crystallization (**Scheme S1B**). After several days the green cubic block crystals of **$\{(\text{L-asp})_2(NiW)_2(BiW_9)_2\}$** were collected. Yield: 364 mg (56 % based on W). Elemental analysis calc (%) for $K_{3.5}Na_{6.5}W_{20}Bi_2Ni_2C_{12}N_3O_{118}H_{103}$ (6676 g mol^{-1}): K 2.0, Na 2.2, W 55.1, Bi 6.3, Ni 1.8, C 2.2, N 0.6, O 28.3, H 1.6; found: K 2.2, Na 3.7, W 52.5, Bi 6.4, Ni 1.7, C 1.7, N 0.7, O 25.9, H 1.2.

2.3 Preparation of $K_4Na_6[\{Ni(gly)(H_2O)_2\}_2(WO_2)_2(B\text{-}\beta\text{-}BiW_9O_{33})_2]\cdot 86H_2O$ $\{(gly)_2(NiW)_2(BiW_9)_2\}$

A sample of $Na_2WO_4\cdot 2H_2O$ (0.66 g, 2mmol) was dissolved in 20 ml NaOAc/HOAc 1 M buffer solution at pH 5.5. $Bi(NO_3)_3\cdot 5H_2O$ (0.14 g, 0.3 mmol), glycine (0.14 g, 0.2 mmol) and KCl (0.15 g, 2 mmol) were added to the sodium tungstate solution. The reaction mixture was heated to 90°C and stirred for 20 min. Then $Ni(NO_3)_2\cdot 6H_2O$ (0.49 g, 1.6 mmol) was added to the solution. The reaction mixture was heated to 90°C, stirred for 60 min, cooled to room temperature, filtered, and kept at room temperature for crystallization (**Scheme S1C**). After several days light green crystals of $K_4Na_6[\{Ni(gly)(H_2O)_2\}_2(WO_2)_2(B\text{-}\beta\text{-}BiW_9O_{33})_2]\cdot 86H_2O$ were collected. Yield: 353 mg (48 % based on W). Elemental analysis calc (%) for $K_4Na_6W_{20}Bi_2Ni_2C_4N_2O_{162}H_{184}$ (7360 g mol^{-1}): K

2.1, Na 1.8, W 49.9, Bi 5.6, Ni 1.6, C 0.6, N 0.4, O 35.1, H 2.5; found: K 2.8, Na 2.1, W 53.4, Bi 6.9, Ni 2.5, C 1.0, N 0.5, O 26.7, H 1.2.

2.4 Preparation of $K_2Na_8[\{Ni(\eta^2\text{-serinol})(H_2O)\}_2\{Ni(H_2O)_2\}_2(B\text{-}\beta\text{-}BiW_9O_{33})_2]\cdot 42H_2O$ $\{(serinol)_2Ni_4(BiW_9)_2\}$

A sample of $Na_2WO_4\cdot 2H_2O$ (0.66 g, 2 mmol) was dissolved in 20 ml NaOAc/HOAc 1M buffer solution at pH 5.5. $Bi(NO_3)_3\cdot 5H_2O$ (0.14 g, 0.3 mmol), serinol (0.18 g, 0.2 mmol) and KCl (0.15 g, 2 mmol) were added to the tungsten solution. The reaction mixture was heated to 90°C and stirred for 20 min. Then $Ni(NO_3)_2\cdot 6H_2O$ (0.49 g, 1.6 mmol) was added to the solution. The reaction mixture was heated again to 90°C and stirred for another 60 min, then cooled to room temperature, filtered, and kept at room temperature for crystallization (**Scheme S1C**). After several days the light green crystals of $K_2Na_8[\{Ni(\eta^2\text{-serinol})(H_2O)\}_2\{Ni(H_2O)_2\}_2(B\text{-}\beta\text{-}BiW_9O_{33})_2]\cdot 42H_2O$ were collected. Yield: 203 mg (29 % based on W). Elemental analysis calc (%) for $K_2Na_8W_{18}Bi_2Ni_4C_6N_2O_{118}H_{103}$ (6326 g mol^{-1}): K 1.2, Na 2.9, W 52.3, Bi 6.6, Ni 3.7, C 1.1, N 0.4, O 29.9, H 1.6; found: K 1.4, Na 4.7, W 49.8, Bi 6.3, Ni 2.2, C 2.1, N 0.2, O 27.8, H 1.4.

3. IR Spectra

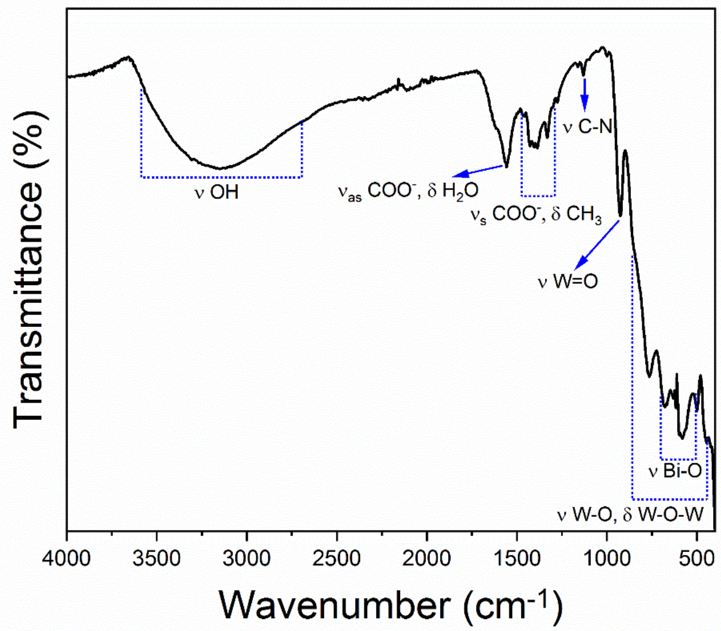

Figure S2. IR spectrum of **{(β-ala)$_4$(Ni$_3$)$_2$(BiW$_9$)$_2$}** from 4000 to 400 cm^{-1}. For peak assignments see Table S1.

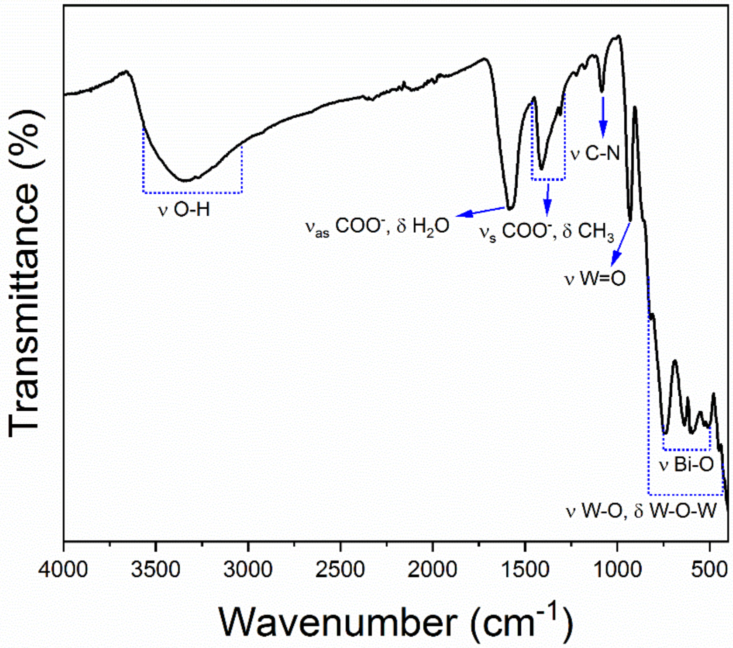

Figure S3. IR spectrum of **{(L-asp)$_2$(NiW)$_2$(BiW$_9$)$_2$}** from 4000 to 400 cm^{-1}. For peak assignments see Table S1.

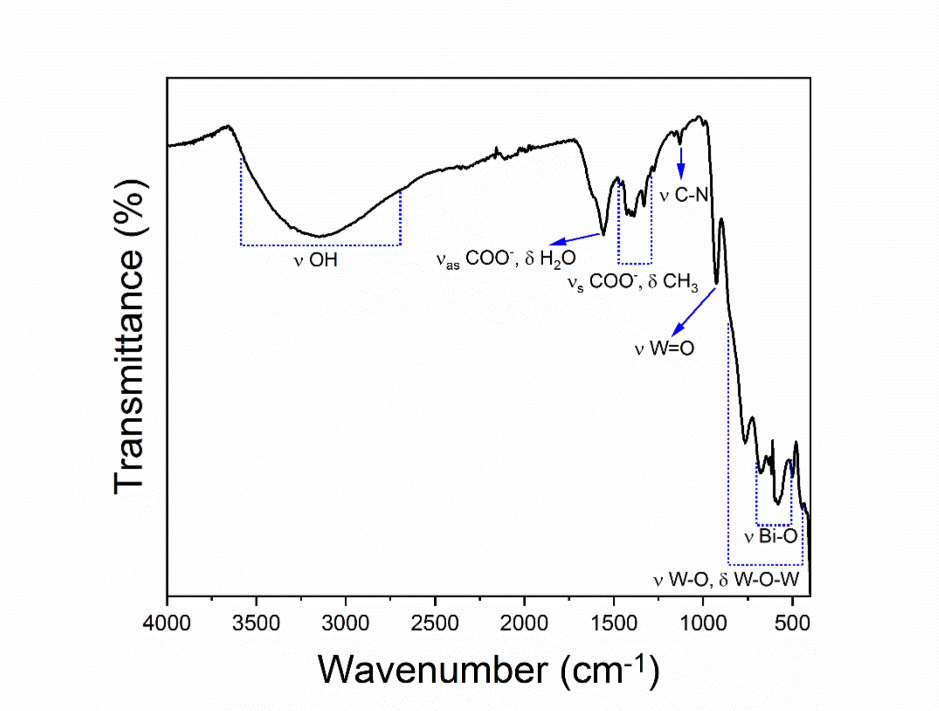

Figure S4. IR spectrum of **{$(gly)_2(NiW)_2(BiW_9)_2$}** from 4000 to 400 cm^{-1}. For peak assignments see Table S1.

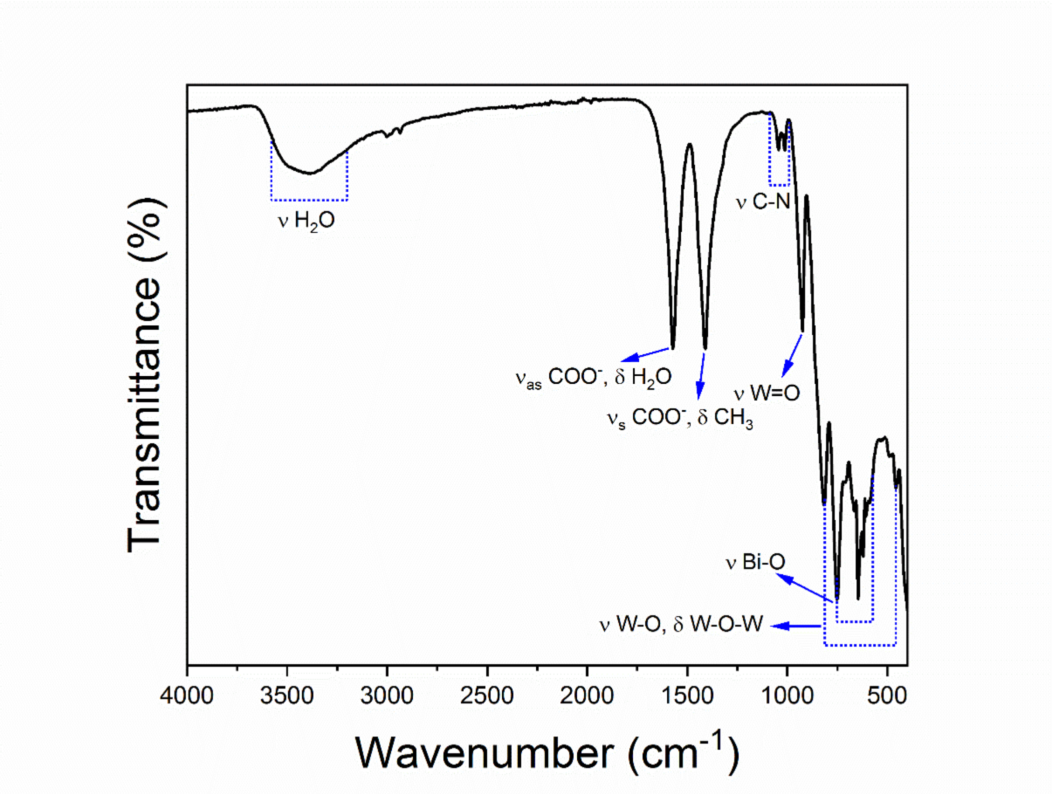

Figure S5. IR spectrum **{$(serinol)_2Ni_4(BiW_9)_2$}** from 4000 to 400 cm^{-1}. For peak assignments see Table S2.

Table S1. Attribution and positions of the bands observed in the IR-spectra based on references (Ref.).

Compound	attribution	Position / range [cm^{-1}]	intensity	Ref.
**{(*β*-ala)$_4$(Ni$_3$)$_2$(BiW$_9$)$_2$}**	ν O-H	3143	m	12
	δ H_2O, ν COO^-	1560	m	
	ν W=O	922	m	
	ν W-O, δ W-O-W	863 - 450	s	
	ν Bi-O	764 - 502	s	13
	δ CH_3, ν COO^-	1437 - 1321	m	14
	ν C-N	1130	w	
**{(L-asp)$_2$(NiW)$_2$(BiW$_9$)$_2$}**	ν O-H	3345	m	13
	δ H_2O, ν COO^-	1581	m	
	ν W=O	930	m	
	ν W-O, δ W-O-W	825 - 430	s	
	ν Bi-O	744 - 572	s	14
	δ CH_3, ν COO^-	1410, 1306	m	15
	ν C-N	1082	w	
**{(gly)$_2$(NiW)$_2$(BiW$_9$)$_2$}**	ν O-H	3329	m	13
	δ H_2O, ν COO^-	1627	m	
	ν W=O	928	m	
	ν W-O, δ W-O-W	807 - 440	s	
	ν Bi-O	755 - 508	s	14
	δ CH_3, ν COO^-	1524 - 1329	m	15
	ν C-N	1105	w	
**{(serinol)$_2$Ni$_4$(BiW$_9$)$_2$}**	ν O-H	3386	m	13
	δ H_2O, ν COO^-	1575	m	
	ν W=O	922	m	
	ν W-O, δ W-O-W	819 - 450	s	
	ν Bi-O	750 - 577	s	14
	δ CH_3, ν COO^-	1413	m	15
	ν C-N	1042, 1013	w	

4. Thermogravimetric analysis

Table S2. Weight-loss (%) values and corresponding water molecules and ligand in all compounds.

Step	T, (°C)	Weight-loss (%)	Number of H_2O and ligand molecules corresponding to mass-loss in compound:
**{(β-ala)$_4$(Ni$_3$)$_2$(BiW$_9$)$_2$}**			
**I**	34-97	9.43	35.5 H_2O
**II**	98-355	7.01	21.5 H_2O + 1 β-alanine
**III**	356-450	1.03	1 β-alanine
**IV**	451-691	2.89	2 β-alanine
**{(L-asp)$_2$(NiW)$_2$(BiW$_9$)$_2$}**			
**I**	25-111	6.11	22 H_2O
**II**	112-120	0.85	3 H_2O
**III**	121-630	7.95	7 H_2O + 3 L-aspartic acid
**{(gly)$_2$(NiW)$_2$(BiW$_9$)$_2$}**			
**I**	23-104	7.18	29.5 H_2O
**II**	105-112	0.99	4 H_2O
**III**	113-371	2.69	11 H_2O
**IV**	372-421	13.12	45.5 H_2O + 2 glycine
**{(serinol)$_2$Ni$_4$(BiW$_9$)$_2$}**			
**I**	25-104	8.84	31 H_2O
**II**	105-132	1.09	4 H_2O
**III**	133-312	2.01	7 H_2O
**IV**	313-528	4.46	6 H_2O + 2 serinol

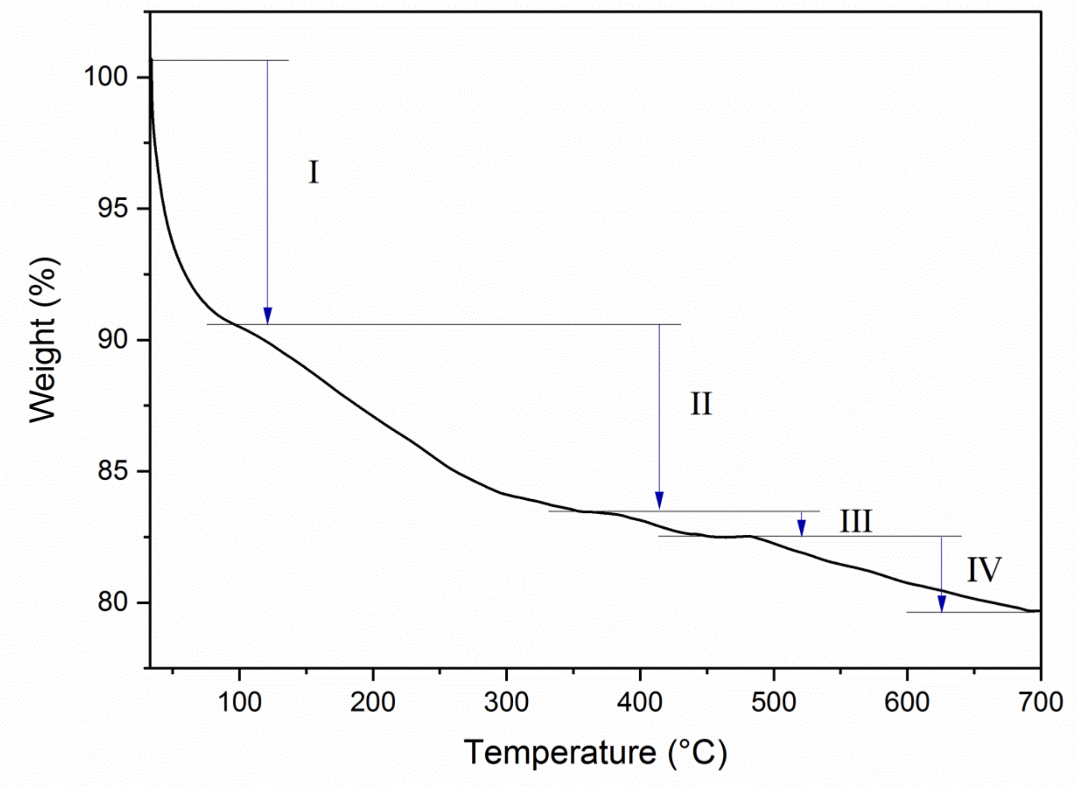

Figure S6. Thermogravimetric curve of **{(*β*-ala)$_4$(Ni$_3$)$_2$(BiW$_9$)$_2$}** with heating rate of 5 °C min^{-1} in the region 25–700 °C. See Table S2 for details.

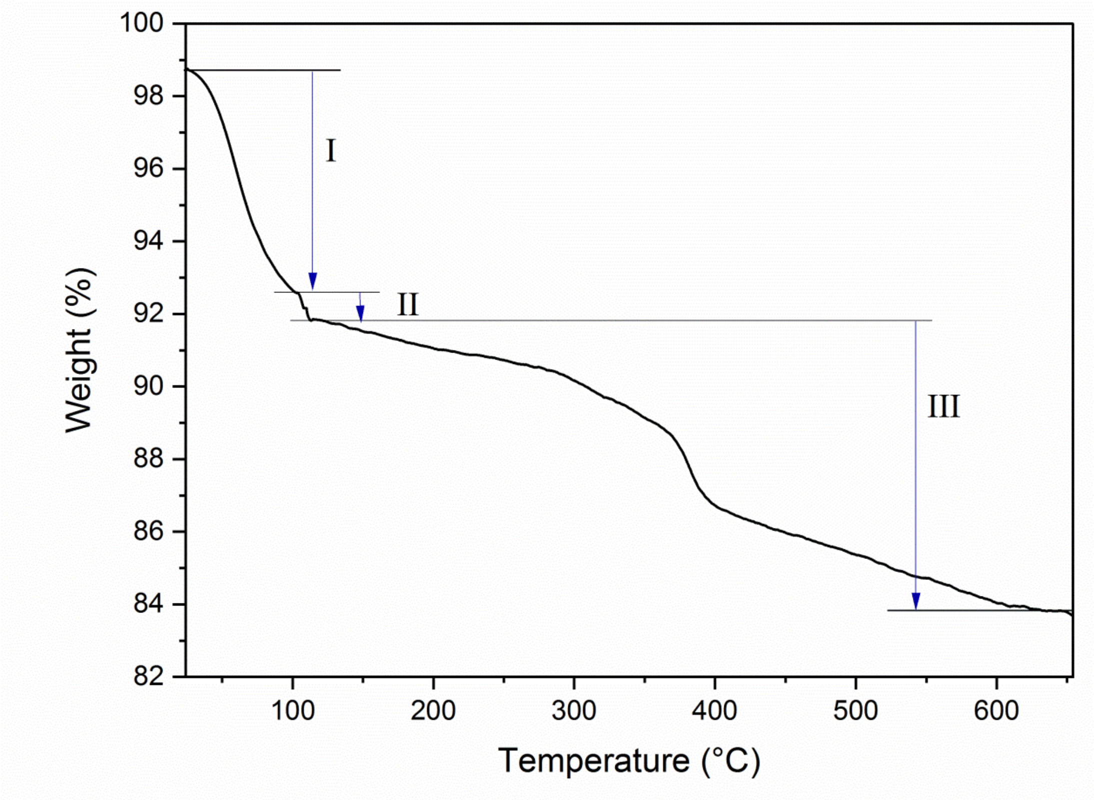

Figure S7. Thermogravimetric curve of **{(L-asp)$_2$(NiW)$_2$(BiW$_9$)$_2$}** with heating rate of 5 °C min^{-1} in the region 25–700 °C. See Table S2 for details.

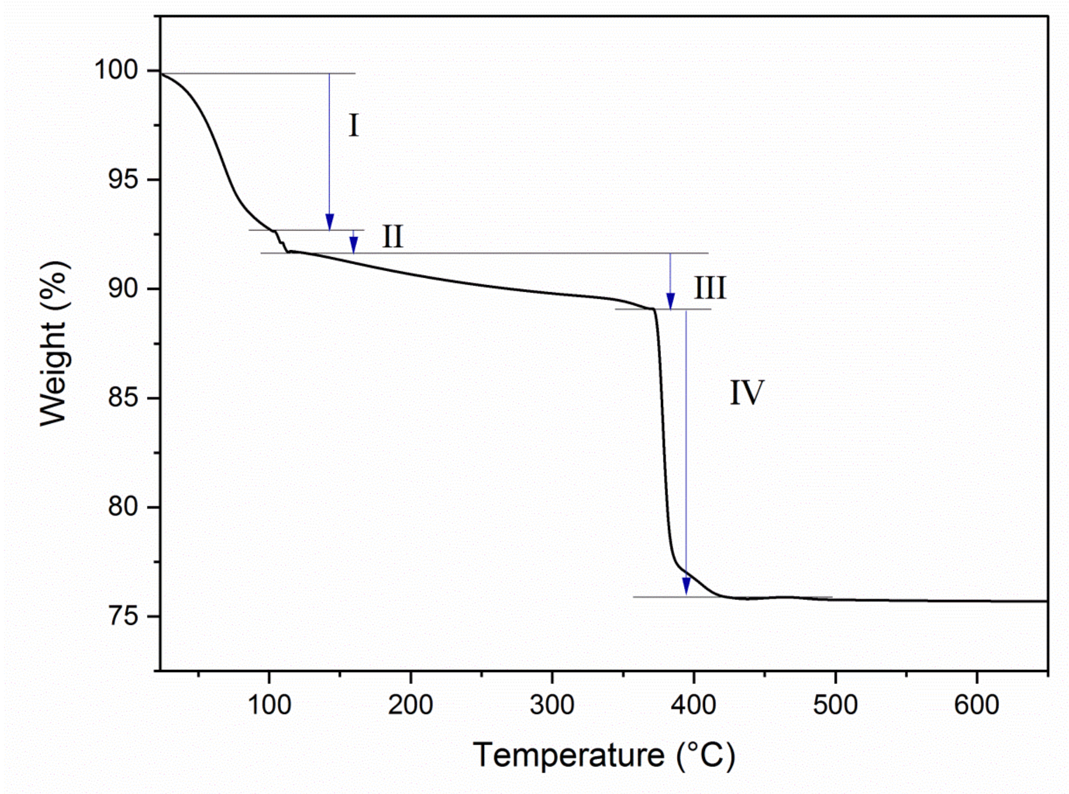

Figure S8. Thermogravimetric curve of **{(gly)$_2$(NiW)$_2$(BiW$_9$)$_2$}** with heating rate of 5 °C min^{-1} in the region 25–700 °C. See Table S2 for details.

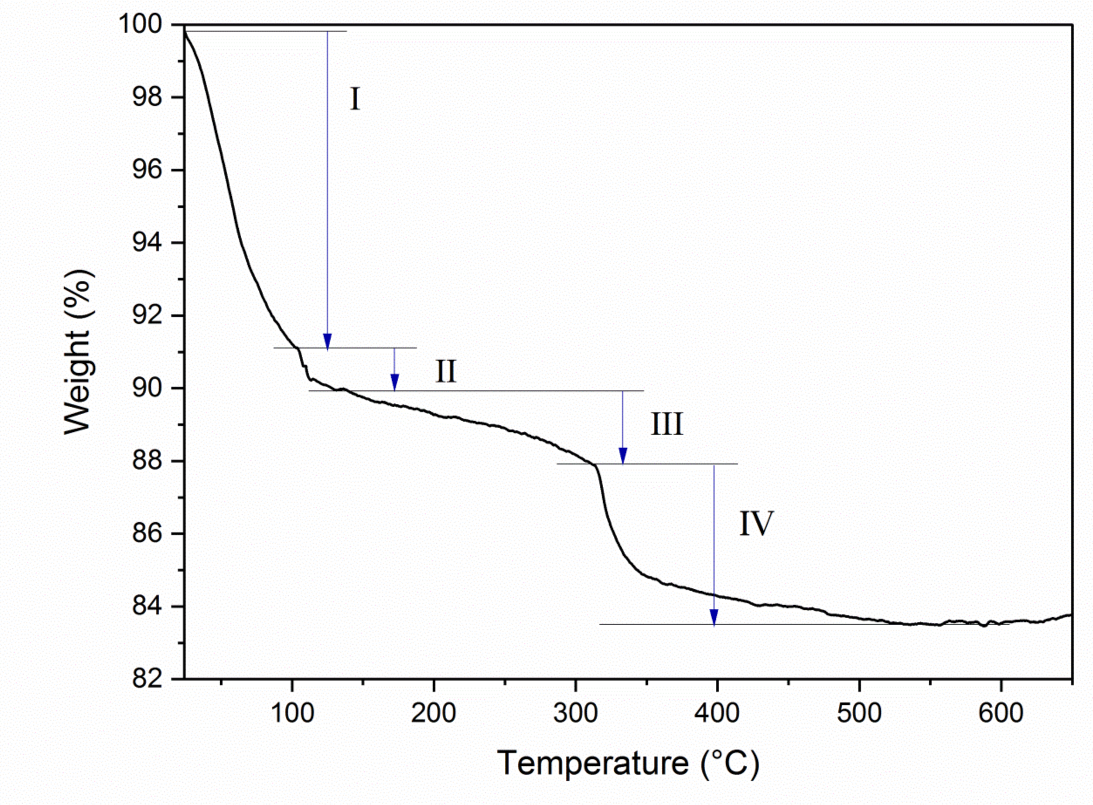

Figure S9. Thermogravimetric curve of **{(serinol)$_2$Ni$_4$(BiW$_9$)$_2$}** with heating rate of 5 °C min^{-1} in the region 25–700 °C. See Table S2 for details.

5. Single-Crystal X-ray Diffraction

Table S3. CCDC-Codes for all compounds.

Sample	CCDC code
**{(β-ala)$_4$(Ni$_3$)$_2$(BiW$_9$)$_2$}**	2246057
**{(L-asp)$_2$(NiW)$_2$(BiW$_9$)$_2$}**	2246055
**{(gly)$_2$(NiW)$_2$(BiW$_9$)$_2$}**	2246054
**{(serinol)$_2$Ni$_4$(BiW$_9$)$_2$}**	2246056

Table S4. Sample and crystal data of **{(β-ala)$_4$(Ni$_3$)$_2$(BiW$_9$)$_2$}**.

**Chemical formula**	$K_4W_{18}Bi_2Ni_8C_{12}N_4O_{131}H_{136}$	**Crystal system**	triclinic	
**Formula weight [g/mol]**	6786	**Space group**	$P\bar{1}$	
**Temperature [K]**	200	**Z**	1	
**Measurement method**	ω scans	**Volume [Å^3]**	3242.5(7)	
**Radiation (Wavelength [Å])**	MoKα (λ = 0.71073)	**Unit cell dimensions [Å] and [°]**	13.1186(16)	90.474(3)
**Crystal size / [mm^3]**	0.2 × 0.15 × 0.125		15.3185(19)	95.053(3)
**Crystal habit**	clear green block		17.239(2)	109.877(3)
**Density (calculated) / [g/cm^3]**	3.272	**Absorption coefficient / [mm^{-1}]**	19.957	
**Abs. correction Tmin**	0.5616	**Abs. correction Tmax**	0.7536	
**Abs. correction type**	multi-scan	**F(000) [e$^-$]**	2816.0	

Table S5. Data collection and structure refinement of **{(β-ala)$_4$(Ni$_3$)$_2$(BiW$_9$)$_2$}**.

**Index ranges**	$-15 \leq h \leq 15$, $-18 \leq k \leq 18$, $-20 \leq l \leq 20$	**Theta range for data collection [°]**	3.548 to 50.7	
**Reflections number**	57760	**Data / restraints / parameters**	11866/78/777	
**Refinement method**	Least squares	**Final R indices**	all data	$R_1 = 0.0520$, $wR_2 = 0.1041$
**Function minimized**	$\Sigma\ w(F_o^2 - F_c^2)^2$		I>2σ(I)	$R_1 = 0.0418$, $wR_2 = 0.0995$
**Goodness-of-fit on F^2**	1.093	**Weighting scheme**	$w=1/[\sigma^2(F_o^2)+(0.0257P)^2+144.9736P]$	
**Largest diff. peak and hole [e Å^{-3}]**	3.47/-2.32		where $P=(F_o^2+2F_c^2)/3$	

Table S6. Sample and crystal data of **{(L-asp)$_2$(NiW)$_2$(BiW$_9$)$_2$}**.

**Chemical formula**	$K_{3.5}Na_{6.5}W_{20}Bi_2Ni_2C_{12}N_3O_{118}H_{103}$	**Crystal system**	monoclinic	
**Formula weight [g/mol]**	6572.50	**Space group**	$P2_1$	
**Temperature [K]**	200	**Z**	2	
**Measurement method**	ω scans	**Volume [Å^3]**	5426.3(7)	
**Radiation (Wave-length [Å])**	MoKα (λ = 0.71073)	**Unit cell dimen-sions [Å] and [°]**	11.9008(8)	90
**Crystal size / [mm^3]**	0.175 × 0.065 × 0.015		29.459(2)	110.942(2)
**Crystal habit**	clear green block		16.5723(12)	90
**Density (calculated) / [g/cm^3]**	4.023	**Absorp-tion co-efficient / [mm^{-1}]**	24.960	
**Abs. correction Tmin**	0.3911	**Abs. cor-rection Tmax**	0.7461	
**Abs. correction type**	multi-scan	**F(000) [e$^-$]**	5754.0	

Table S7. Data collection and structure refinement of **{(L-asp)$_2$(NiW)$_2$(BiW$_9$)$_2$}**.

**Index ranges**	-14 ≤ h ≤ 14, -35 ≤ k ≤ 35, -19 ≤ l ≤ 19	**Theta range for data collection [°]**	2.632 to 50.698	
**Reflections num-ber**	127165	**Data / restraints / parameters**	19871/159/1476	
**Refinement method**	Least squares	**Final R indices**	all data	R_1 = 0.0231, wR_2 = 0.0541
**Function mini-mized**	$\Sigma\ w(F_o^2 - F_c^2)^2$		I>2σ(I)	R_1 = 0.0218, wR_2 = 0.0535
**Goodness-of-fit on F^2**	1.049	**Weighting scheme**	$w=1/[\sigma^2(F_o^2)+(0.0194P)^2+68.9597P]$	
**Largest diff. peak and hole [e Å^{-3}]**	2.318/-1.717		where $P=(F_o^2+2F_c^2)/3$	
**Flack**	0.048(5)			

Table S8. Sample and crystal data of **$\{(gly)_2(NiW)_2(BiW_9)_2\}$**.

**Chemical formula**	$K_4Na_6W_{20}Bi_2Ni_2C_4N_2O_{162}H_{184}$	**Crystal system**	monoclinic	
**Formula weight [g/mol]**	7360	**Space group**	*C*2/*m*	
**Temperature [K]**	200	**Z**	2	
**Measurement method**	ω scans	**Volume [Å^3]**	4962.2(15)	
**Radiation (Wavelength [Å])**	MoKα (λ = 0.71073)	**Unit cell dimensions [Å] and [°]**	16.135(3)	90
**Crystal size / [mm^3]**	0.2 × 0.175 × 0.175		18.221(3)	101.976(5)
**Crystal habit**	clear green block		17.254(3)	90
**Density (calculated) / [g/cm^3]**	4.128	**Absorption coefficient / [mm^{-1}]**	26.539	
**Abs. correction Tmin**	0.5616	**Abs. correction Tmax**	0.7536	
**Abs. correction type**	multi-scan	**F(000) [e$^-$]**	5374.0	

Table S9. Data collection and structure refinement of **$\{(gly)_2(NiW)_2(BiW_9)_2\}$**.

**Index ranges**	$-18 \le h \le 18$, $-21 \le k \le 21$, $-20 \le l \le 20$	**Theta range for data collection [°]**	3.414 to 48.812	
**Reflections number**	56261	**Data / restraints / parameters**	4220/60/397	
**Refinement method**	Least squares	**Final R indices**	all data	R_1 = 0.0544, wR_2 = 0.1461
**Function minimized**	$\Sigma\ w(F_o^2 - F_c^2)^2$		I>2σ(I)	R_1 = 0.0526, wR_2 = 0.1434
**Goodness-of-fit on F^2**	1.176	**Weighting scheme**	$w=1/[\sigma^2(F_o^2)+(0.1193P)^2+342.5708P]$	
**Largest diff. peak and hole [e Å^{-3}]**	2.71/-1.41		where $P=(F_o^2+2F_c^2)/3$	

Table S10. Sample and crystal data **{(serinol)$_2$Ni$_4$(BiW$_9$)$_2$}**.

**Chemical formula**	$K_2Na_8W_{18}Bi_2Ni_4C_6N_2O_{118}H_{103}$	**Crystal system**	triclinic	
**Formula weight [g/mol]**	6326	**Space group**	$P\bar{1}$	
**Temperature [K]**	200	**Z**	1	
**Measurement method**	ω scans	**Volume [Å^3]**	2407.1(5)	
**Radiation (Wavelength [Å])**	MoKα (λ = 0.71073)	**Unit cell dimen-sions [Å] and [°]**	12.6180(16)	105.058(4)
**Crystal size / [mm^3]**	0.65 × 0.5 × 0.15		12.6510(16)	108.171(4)
**Crystal habit**	clear green block		16.549(2)	91.499(4)
**Density (calculated) / [g/cm^3]**	4.163	**Absorp-tion coef-ficient / [mm^{-1}]**	26.527	
**Abs. correction Tmin**	0.5616	**Abs. cor-rection Tmax**	0.7536	
**Abs. correction type**	multi-scan	**F(000) [e$^-$]**	2641.0	

Table S11. Data collection and structure refinement of **{(serinol)$_2$Ni$_4$(BiW$_9$)$_2$}**.

**Index ranges**	-15 ≤ h ≤ 15, -15 ≤ k ≤ 15, -19 ≤ l ≤ 19	**Theta range for data collection [°]**	3.356 to 50.698	
**Reflections num-ber**	58835	**Data / restraints / parameters**	8812/6/703	
**Refinement method**	Least squares	**Final R indices**	all data	R_1 = 0.0254, wR_2 = 0.0515
**Function mini-mized**	Σ w(F_o^2 - F_c^2)^2		I>2σ(I)	R_1 = 0.0209, wR_2 = 0.0496
**Goodness-of-fit on F^2**	1.053	**Weighting scheme**	w=1/[σ^2(F_o^2)+(0.0145P)^2+29.9331P]	
**Largest diff. peak and hole [e Å^{-3}]**	1.64/-1.39		where P=(F_o^2+2F_c^2)/3	

Table S12. List of nickel bond lengths for all compounds. Bonds between Ni and N/O related to the organic ligands are tagged with *.

**$\{(\beta\text{-ala})_4(Ni_3)_2(BiW_9)_2\}$**		**$\{(\text{L-asp})_2(NiW)_2(BiW_9)_2\}$**		**$\{(\text{gly})_2(NiW)_2(BiW_9)_2\}$**		**$\{(\text{serinol})_2Ni_4(BiW_9)_2\}$**	
Bond	d / Å	Bond	d / Å	Bond	d / Å	Bond	d / Å
Ni(3)-O(14)	2.051(10)	Ni1 O32	2.071(14)	Ni(1)-O(27)*	1.990(14)	Ni(2)-O(24)	2.042(5)
Ni(3)-O(40)	2.072(1)	Ni1 O63	2.052(14)	Ni(1)-O(26)	2.089(11)	Ni(2)-O(27)	2.052(5)
Ni(3)-O(41)*	2.061(11)	Ni1 O66	2.050(13)	Ni(1)-O(26)	2.089(11)	Ni(2)-O(32)	2.013(5)
Ni(3)-O(30)	2.054(10)	Ni1 O71*	2.030(14)	Ni(1)-O(6)	2.058(10)	Ni(2)-O(38)*	2.072(5)
Ni(3)-(O18)	2.038(9)	Ni1 O72*	2.003(14)	Ni(1)-O(6)	2.058(10)	Ni(2)-O(39)	2.085(6)
Ni(3)-O(39)	2.052(10)	Ni1 N1*	2.092(18)	Ni(1)-O(16)	2.009(14)	Ni(2)-N(1)*	2.070(6)
Ni(2)-O(17)	2.015(9)	Ni2 O7	2.032(13)			Ni(1)-O(35)	2.066(6)
Ni(2)-O(31)	2.037(10)	Ni2 O17	2.063(13)			Ni(1)-O(34)	2.077(5)
Ni(2)-O(19)	1.992(10)	Ni2 O42	2.019(14)			Ni(1)-O(8)	2.024(5)
Ni(2)-O(35)*	2.042(9)	Ni2 O73*	2.062(14)			Ni(1)-O(19)	2.028(5)
Ni(2)-O(5)	2.036(10)	Ni2 O74*	2.040(14)			Ni(1)-O(31)	2.022(5)
Ni(2)-O(36)	2.083(9)	Ni2 N2*	2.076(18)			Ni(1)-O(33)	2.013(5)
Ni(1)-O(35)	1.999(9)						
Ni(1)-O(34)	2.162(11)						
Ni(1)-N(1)*	2.047(13)						
Ni(1)-O(10)	2.101(9)						
Ni(1)-O(8)	2.218(10)						
Ni(1)-O(17)	2.067(9)						

6. Powder X-ray Diffraction

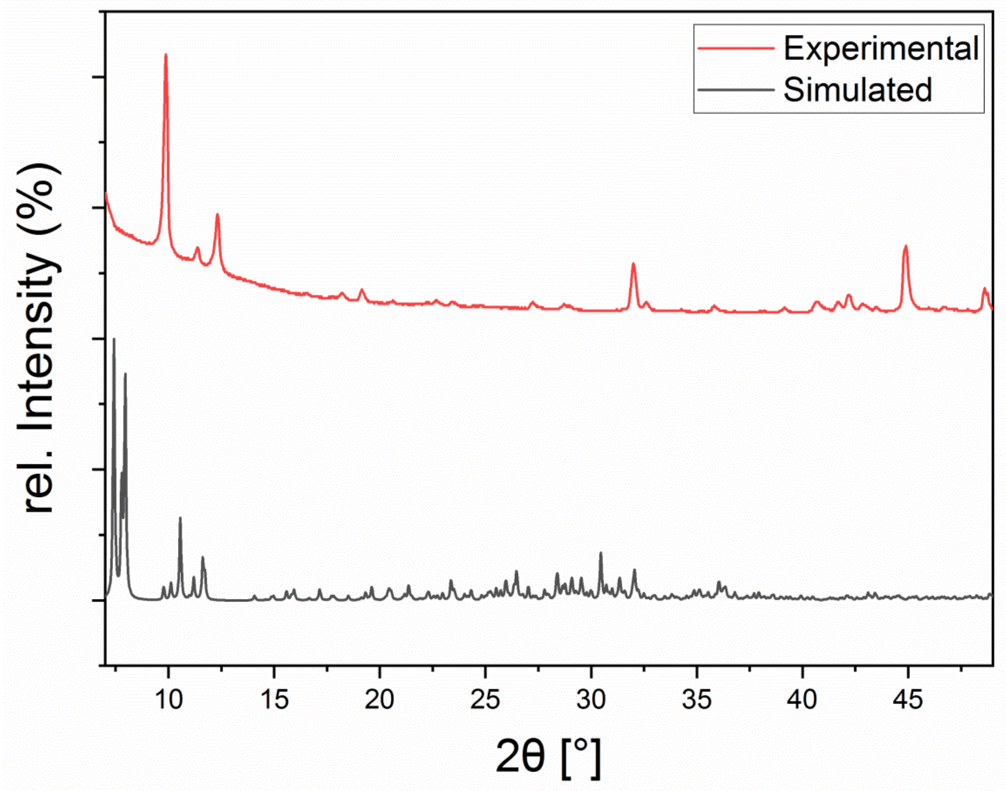

Figure S9. Comparison of the experimental and simulated PXRD patterns of **{(*β*-ala)$_4$(Ni$_3$)$_2$(BiW$_9$)$_2$}**. The difference between the experimental and simulated PXRD pattern could be related to the decomposition of the crystal lattice.

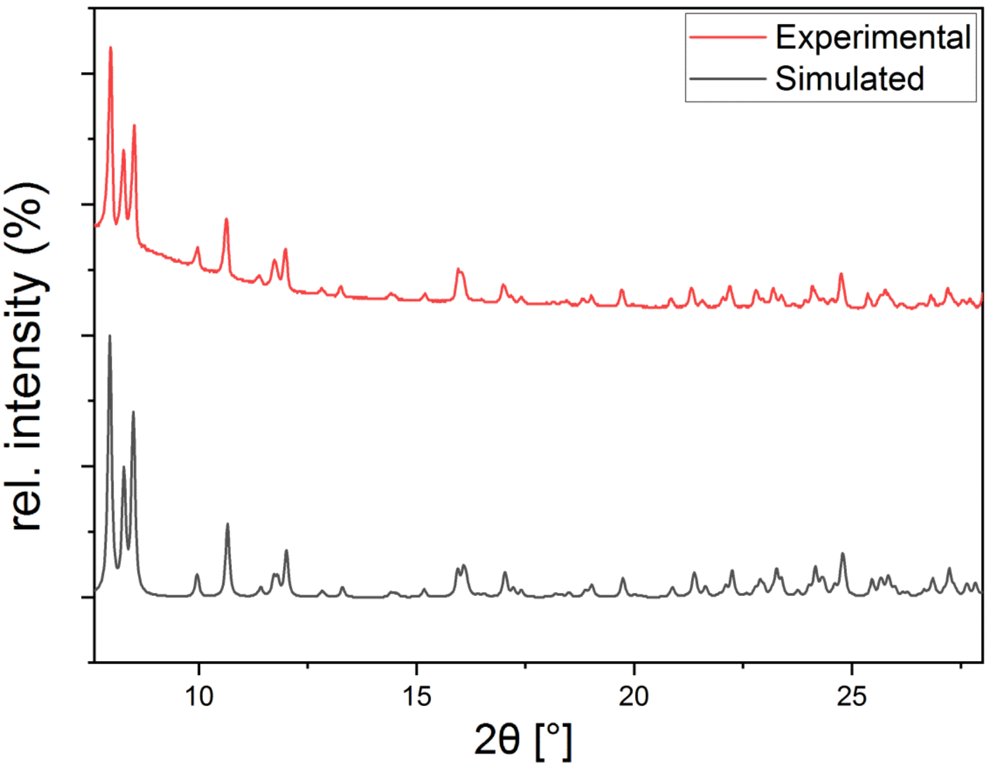

Figure S10. The experimental and simulated PXRD patterns of **{(L-asp)$_2$(NiW)$_2$(BiW$_9$)$_2$}** show the high compatibility of the experimental pattern to the simulated PXRD one**.**

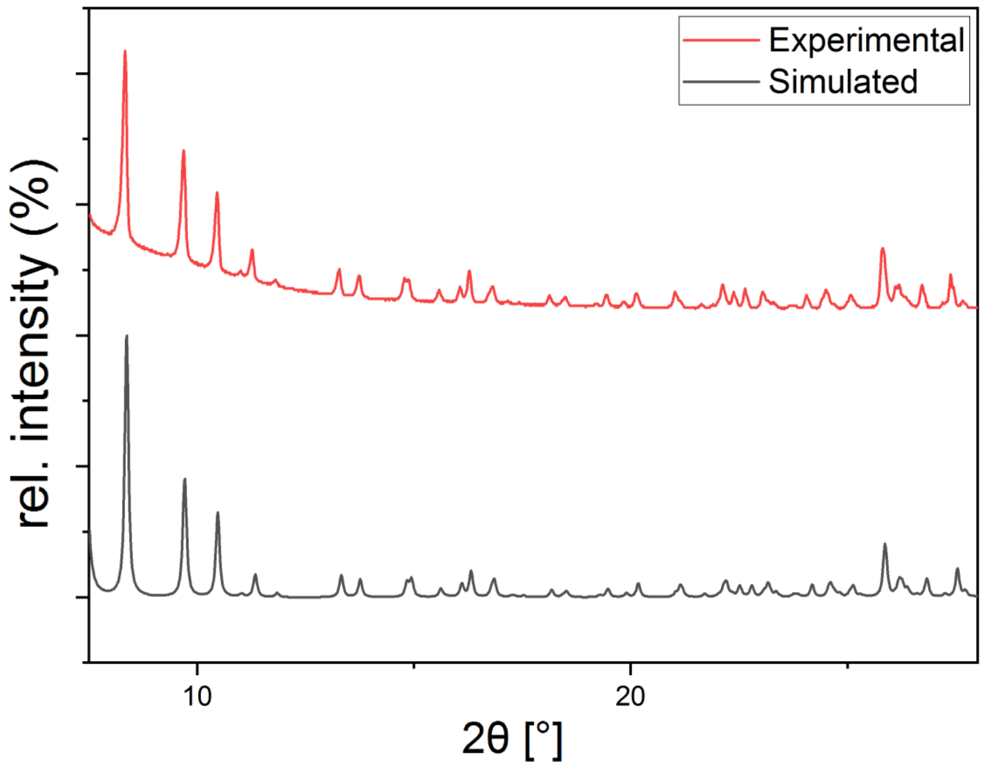

Figure S11. Comparison of the experimental and simulated PXRD patterns of **{(gly)$_2$(NiW)$_2$(BiW$_9$)$_2$}** shows the high compatibility of the experimental pattern to the simulated PXRD one.

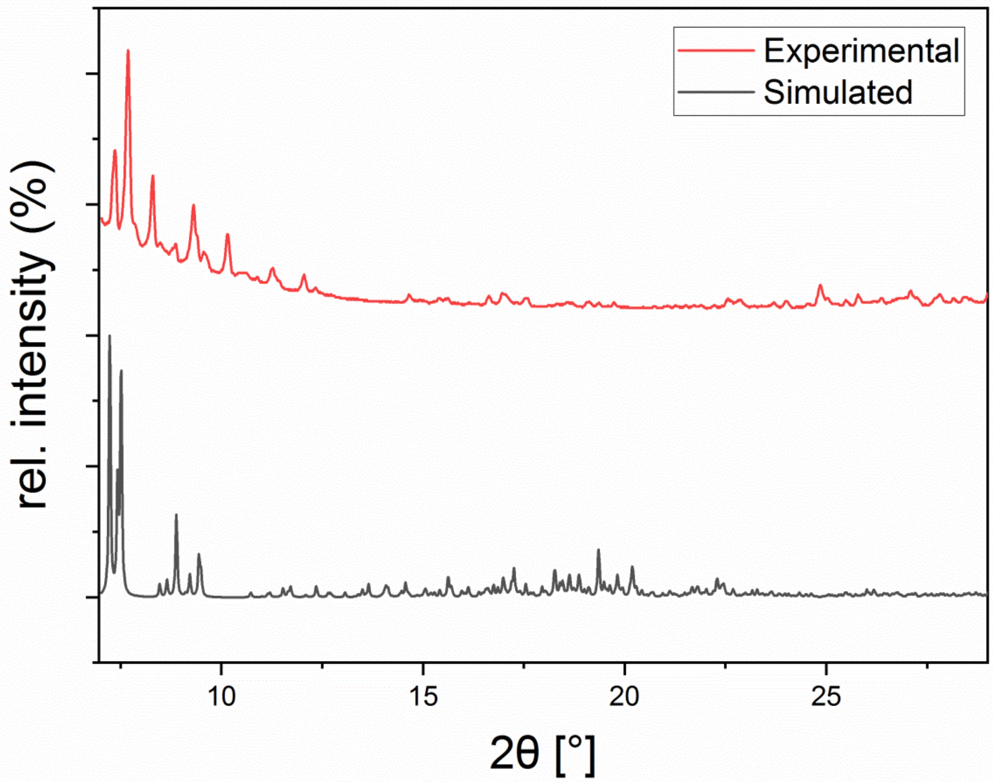

Figure S12. Comparison of the experimental and simulated PXRD patterns of **{(serinol)$_2$Ni$_4$(BiW$_9$)$_2$}**. The difference between experimental and simulated PXRD patterns could be attributed to the crystal lattice decomposition due to water molecules loss.

7. Vis Spectroscopy and ^{1}H NMR spectroscopy

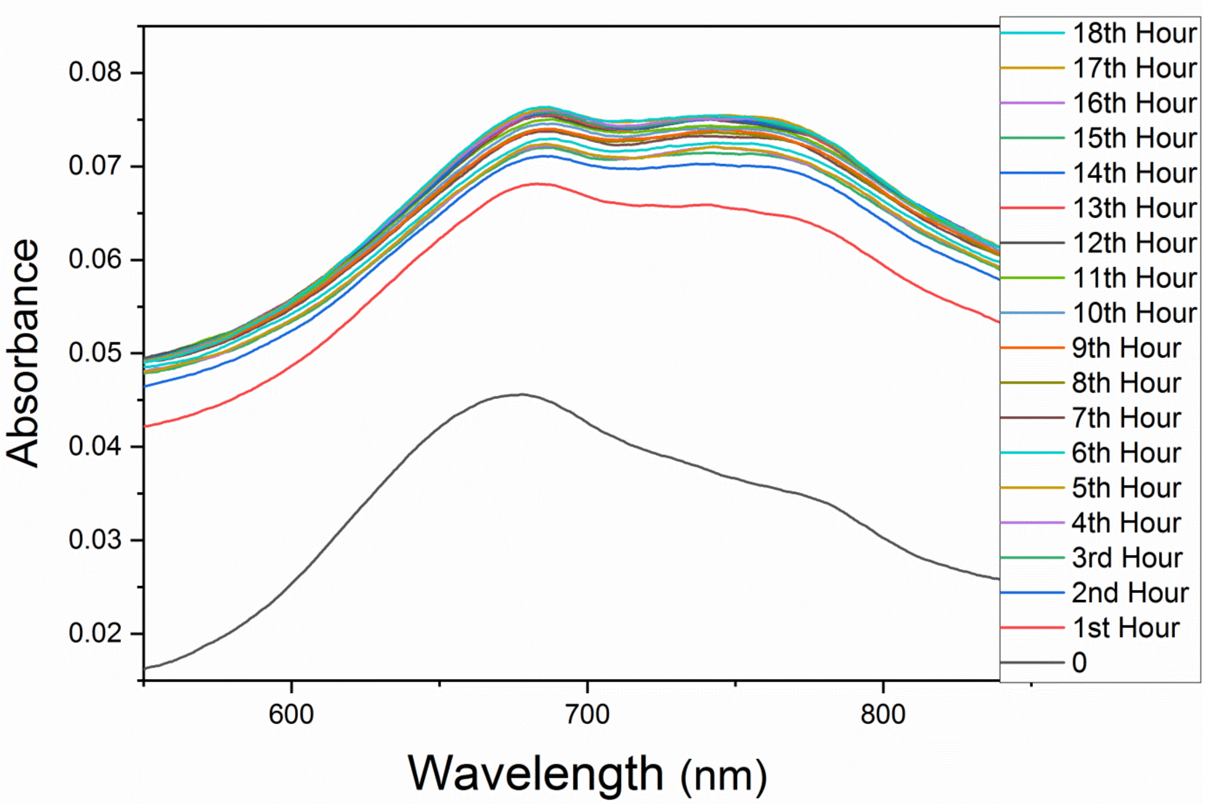

Figure S13. Vis spectrum of [3 mM] **{(L-asp)$_2$(NiW)$_2$(BiW$_9$)$_2$}** in H_2O at 25 °C for 18 hours (Vis absorption was recorded every hour). The growth of a shoulder peak and corresponding rise in absorbance could be attributed to the release and partial hydration of the nickel ion from Krebs-type anion, which is fairly similar to the visible spectrum of nickel nitrate aqueous solution (**Figure S14**).

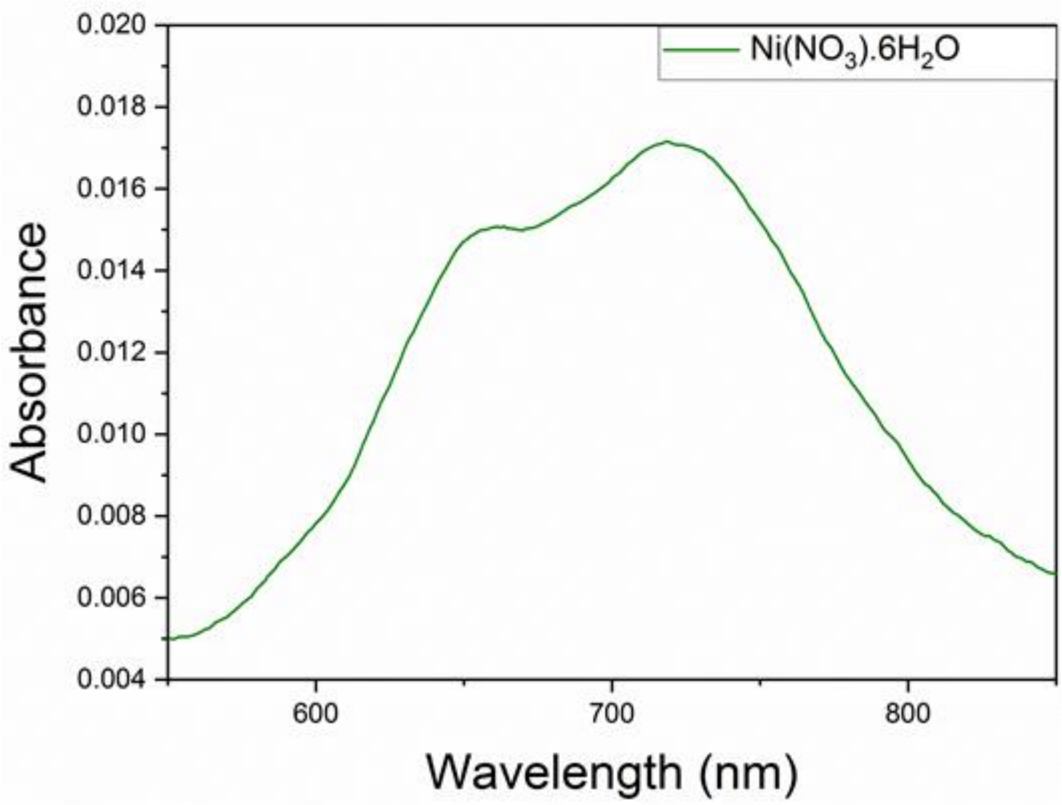

Figure S14. Vis spectra of [6 mM] **$Ni(NO_3)_2·6H_2O$** in H_2O at 25 °C.

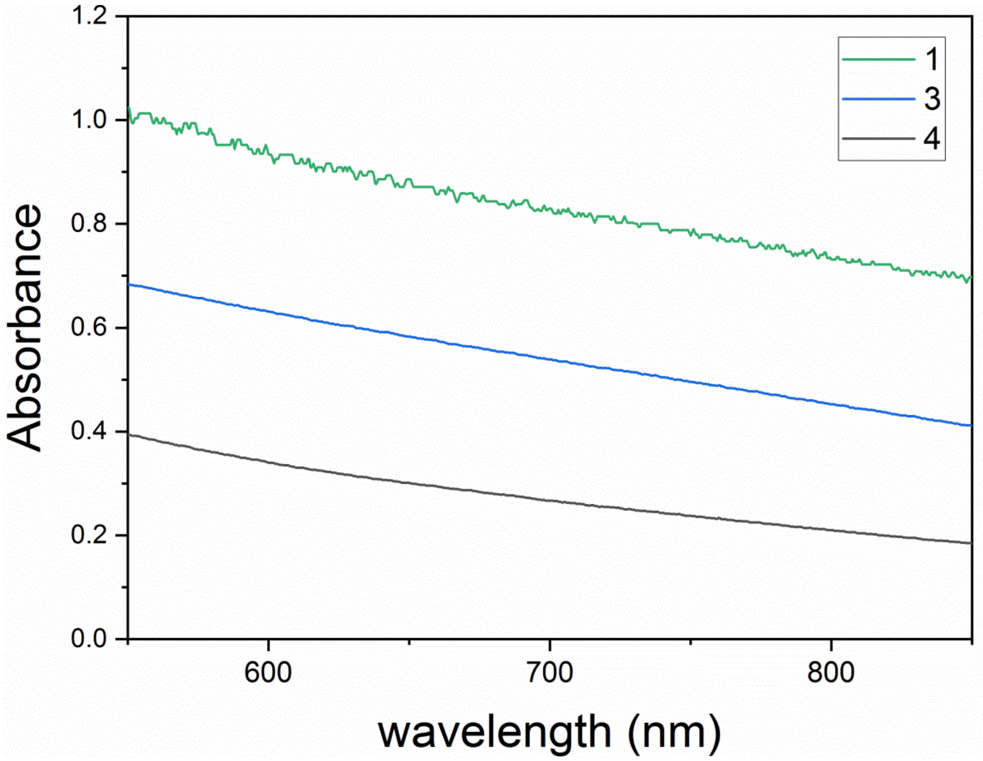

Figure S15. Vis spectra of [0.75 mM] compounds **{(*β*-ala)$_4$(Ni$_3$)$_2$(BiW$_9$)$_2$} (1)**, **{(gly)$_2$(NiW)$_2$(BiW$_9$)$_2$} (3)** and **{(serinol)$_2$Ni$_4$(BiW$_9$)$_2$} (4)** in H_2O at 25 °C.

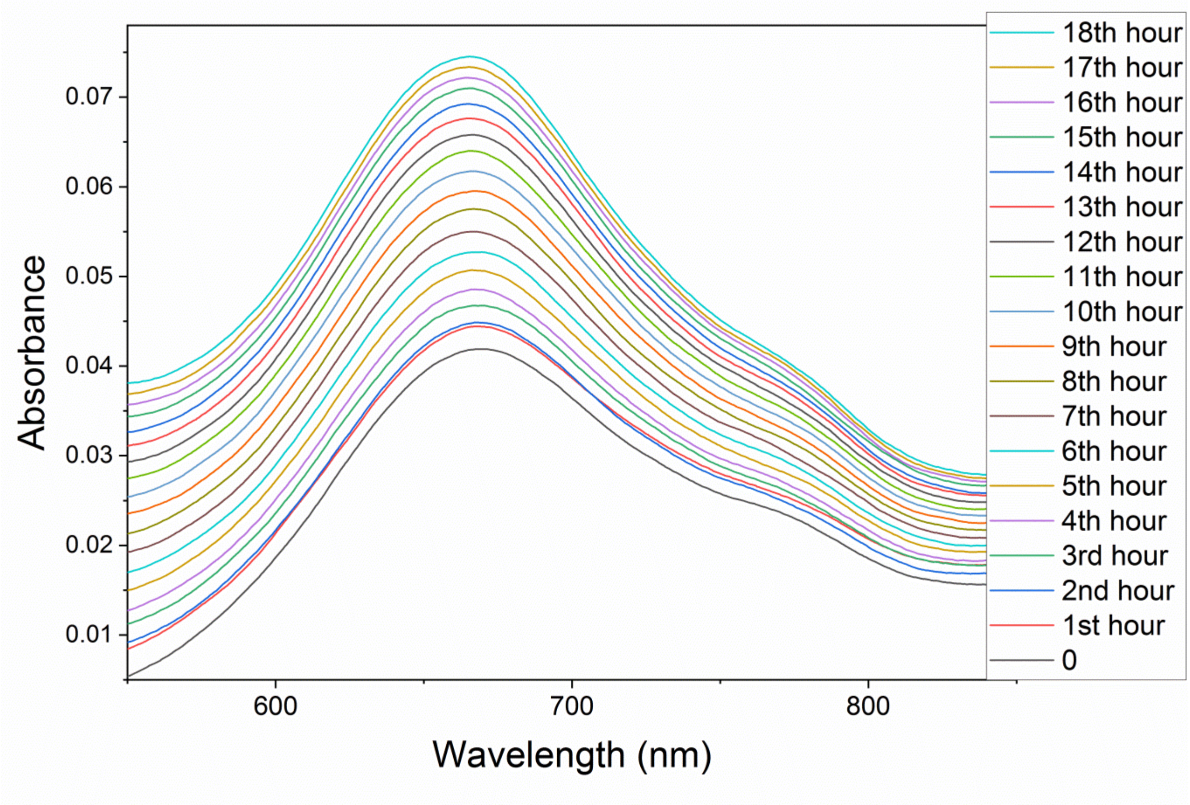

Figure S16. Vis spectra of [3 mM] **{(L-asp)$_2$(NiW)$_2$(BiW$_9$)$_2$}** in Mueller Hinton Broth[15] medium for MIC-determination for 18 hours at 37 °C (Vis absorption was recorded every hour). The absorption increase could be attributed to the dissociation of L-aspartic acid from Krebs-type anion or a change in its chelation mode towards nickel ion, which changes the nickel ion coordination environment and absorption.

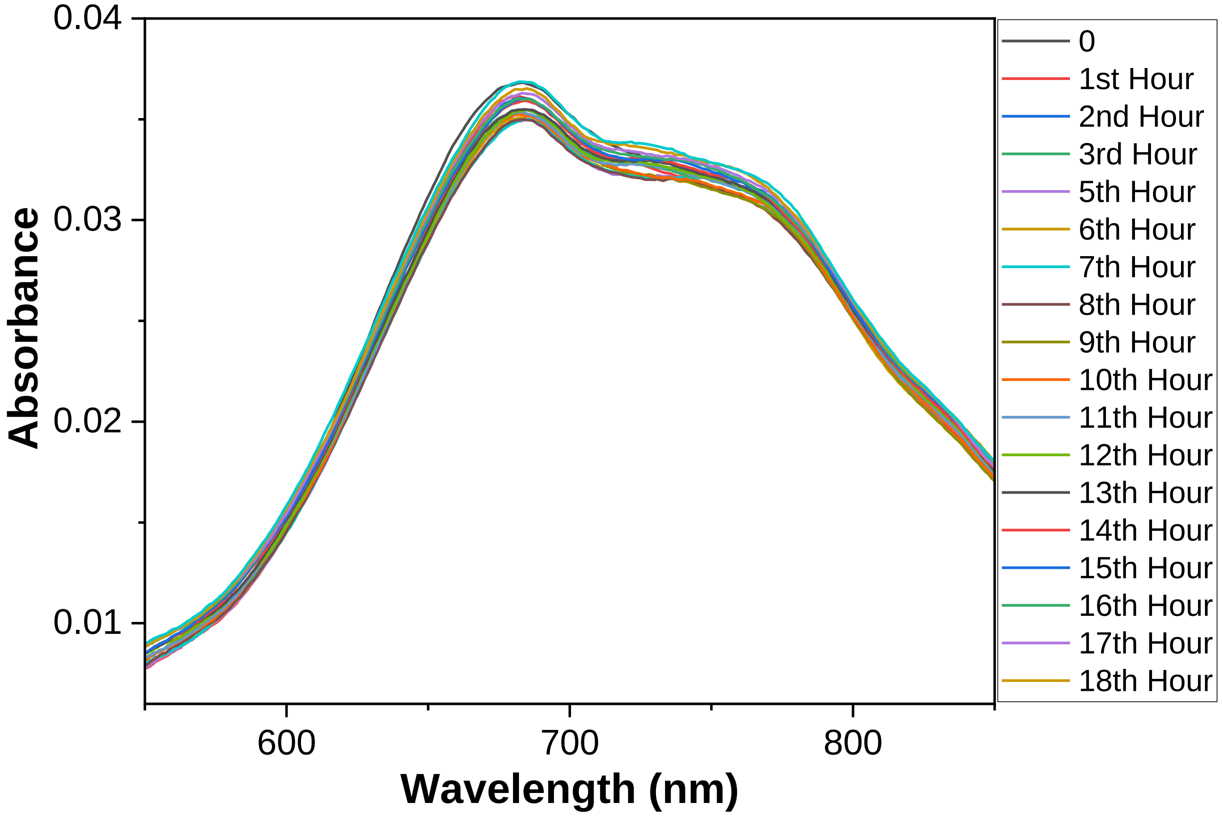

Figure S17. Vis spectra of [3 mM] **$\{(L\text{-}asp)_2(NiW)_2(BiW_9)_2\}$** in 50 mM HEPES buffer pH 7.4 for 18 hours (Vis absorption was recorded every hour). The absorption almost does not change over time, which might indicate the stability of the compound under the conditions applied.

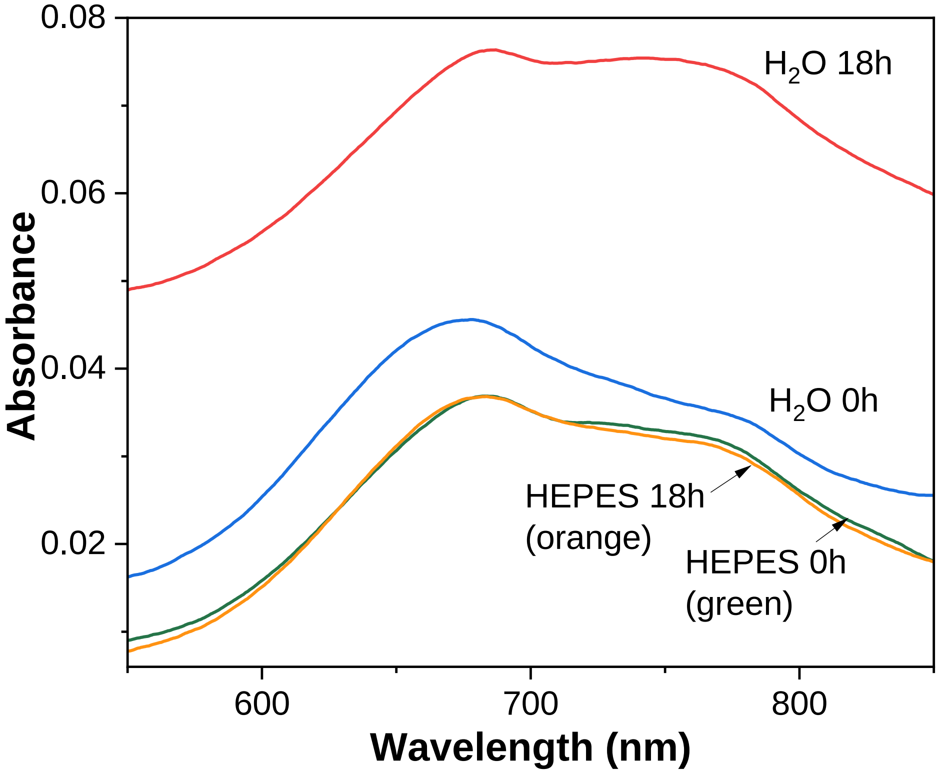

Figure S18. Vis spectra of [3 mM] **{(L-asp)$_2$(NiW)$_2$(BiW$_9$)$_2$}** in H_2O and in 50 mM HEPES buffer; pH 7.4 at 25 °C.

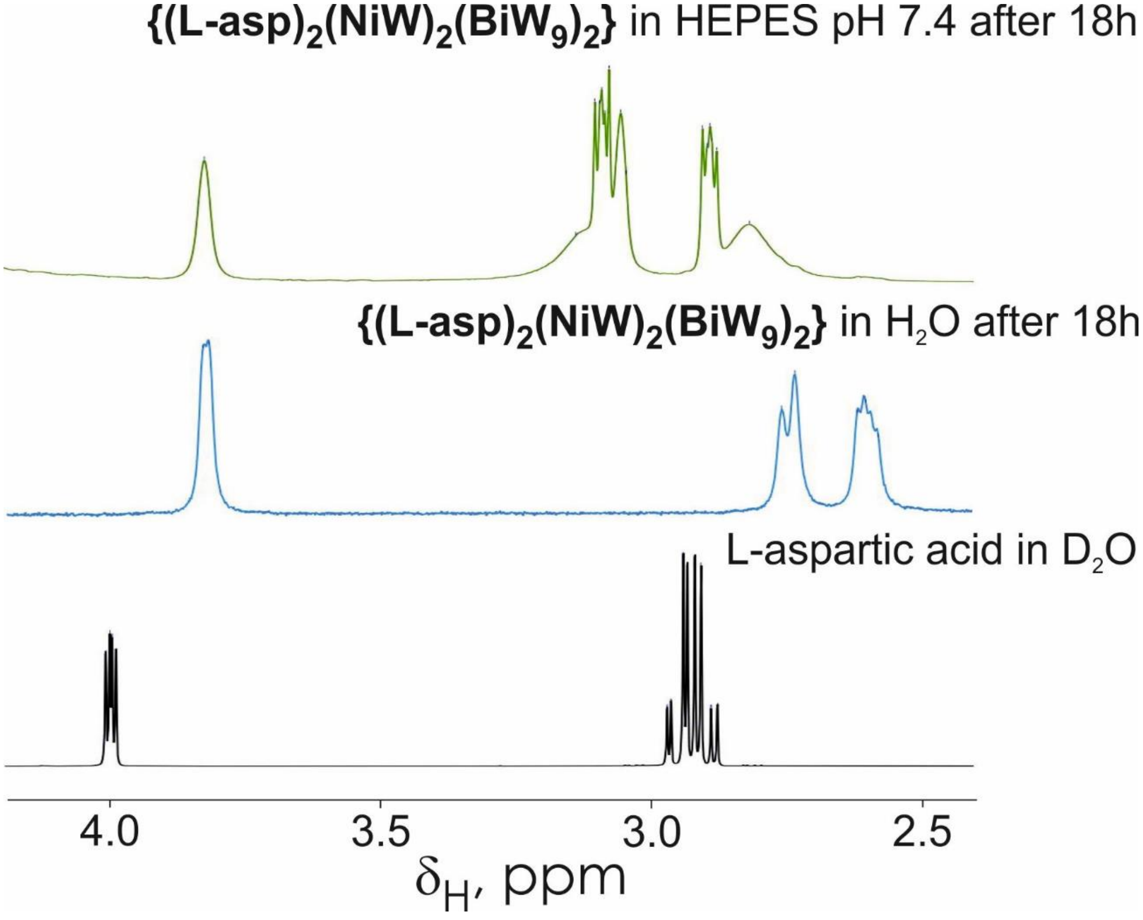

Figure S19. ^{1}H NMR spectra of L-aspartic acid recorded immediately after preparation and **{(L-asp)$_2$(NiW)$_2$(BiW$_9$)$_2$}** in H_2O and in 50 mM HEPES buffer with pH 7.4 at 25 °C recorded 18 hours after preparation. The presence of paramagnetic Ni(II) ion causes paramagnetic relaxation enhancement leading to line broadening and signal suppression in the proton NMR spectra.

8. Antibacterial activity

Table S13. Details on the investigated compounds and minimum inhibitory concentration (MIC) of **{(*β*-ala)$_4$(Ni$_3$)$_2$(BiW$_9$)$_2$}**, **{(L-asp)$_2$(NiW)$_2$(BiW$_9$)$_2$}**, **{(gly)$_2$(NiW)$_2$(BiW$_9$)$_2$}**, **{(serinol)$_2$Ni$_4$(BiW$_9$)$_2$}, {(NiW)$_2$(BiW$_9$)$_2$}** against *Staphylococcus aureus* (***S. aureus*-ATCC 13709**), *Enterococcus faec*alis (***E. faecalis*-ATCC29212**), *Moraxella catarrhalis* (***M. catarrhalis*-ATCC 23246**) and *Escherichia coli* (***E. coli*-TolC-Tn10**).

**Compound**	**c (mg/ml)**	**c (mM)**	**dissolving properties in H_2O**	**MIC (µg/ml)**			
				***S. aureus* ATCC 13709**	***E. faecalis* ATCC29212**	***M. catarrhalis* ATCC 23246**	***E. coli* TolC-Tn10**
**{(*β*-ala)$_4$(Ni$_3$)$_2$(BiW$_9$)$_2$}***	20	2.94	yellow suspension	256	32	8	>256
**{(L-asp)2(NiW)2(BiW9)2}**	20	3.07	dissolved	>1024	>1024	128	>1024
**{(gly)$_2$(NiW)$_2$(BiW$_9$)$_2$}***	20	2.72	yellow suspension	>1024	>1024	256	>1024
**{(serinol)$_2$Ni$_4$(BiW$_9$)$_2$}**	20	3.16	milky suspension	>1024	>1024	512	>1024
**{(NiW)$_2$(BiW$_9$)$_2$}***	20	3.23	milky suspension	256	128	128	>1024
**Ni(NO$_3$)$_2$**	10	34.39	dissolved	>256	>256	>256	>256
**Bi(NO$_3$)$_3$***	10	20.62	poorly soluble	64	>256	>256	>256
**β-alanine**	20	224.72	dissolved	>256	>256	>256	>256
**L-Aspartic acid**	20	150.38	dissolved	>256	>256	>256	>256
**glycine**	10	133.33	dissolved	>256	>256	>256	>256
**serinol**	20	219.78	dissolved	>256	>256	>256	>256
**azithromycin**	20	26.70	dissolved	0.5	8	0.125	0.5

* precipitate in wells down to 16 µg/mL

9. References

[1] Rosu, C.; Weakley, T. J. R. Hexasodium tetrahydrogen bis(triaquanickelo)ico-satungstodibismuthate(III)(10-) 36-hydrate. *Acta Cryst.* **1999**, 55 (4), 1-3. DOI: 10.1107/S0108270199099710

[2] Bruker SAINT v7.68A Copyright © **2005–2016** Bruker AXS.

[3] Sheldrick, G. M. SHELXT – Integrated Space-Group and Crystal-Structure Determination. *Acta Cryst A* **2015**, *71* (1), 3–8. DOI: 10.1107/S2053273314026370

[4] Dolomanov, O.V.; Bourhis, L. J.; Gildea, R. J.; Howard, J. A. K.; Puschmann, H. OLEX2: A Complete Structure Solution, Refinement and Analysis Program. *J. Appl. Cryst.* **2009**, 42, 339-341. DOI: 10.1107/S0021889808042726

[5] Macrae, C. F.; Sovago, I. S.; Cottrell, J.; Galek, P. T. A.; McCabe, P.; Pidcock, E.; Platings, M.; Shields, G. P.; Stevens, J. S.; Towler, M.; Wood, P. A. Mercury 4.0: from visualization to analysis, design and prediction. *J. Appl. Cryst.* **2020**, 53, 226-235. DOI: 10.1107/S1600576719014092

[6] Wayne, P. Clinical Laboratory Standard Institute Methods for Dilution Antimicrobial Suscepti-bility Tests f or Bacteria That Grow Aerobically; Approved Standard. M07-A8. *CLSI*. **2009**, 8.

[7] Greenwood, D. Antimicrobial drugs: chronicle of a twentieth century medical triumph. *Oxford University Press* **2008**, 1, 239-241. DOI: 10.1093/jac/dkp066

[8] Wang, J.; Ma, P.; Shen, Y.; Niu, J. Tetra-Transition-Metal Substituted Weakley-Type Sandwich Germanotungstates and their Derivatives Decorated by Transition-Metal Com-plexes. *Cryst. Growth Des.* **2008**, 8 (9), 3130-3133. DOI: 10.1021/cg701278b

[9] Bösing, M.; Loose, I.; Pohlmann, H.; Krebs, B. New Strategies for the Generation of Large Het-eropolymetalate Clusters: The β-B-SbW_9 Fragment as a Multifunctional Unit. *Chem. Eur. J.* **1997**, 3 (8), 1232-1237. DOI: 10.1002/chem.19970030810

[10] Laronze, N.; Marrot, J.; Hervé, G. Cation-Directed Synthesis of Tungstosilicates. 1. Syntheses and Structures of K. Cation-Directed Synthesis of Tungstosilicates. 1. Syntheses and Structures of $K_{10}A$-α-$[SiW_9O_{34}]\cdot 24H_2O$, of the Sandwich-Type Complex $K_{10.75}[Co(H_2O)_6]_{0.5}[Co(H_2O)_4Cl]_{0.25}A$-$\alpha$-$[K_2\{Co(H_2O)_2\}_3(SiW_9O_{34})_2]\cdot 32H_2O$ and of $Cs_{15}[K(SiW_{11}O_{39})_2]\cdot 39H_2O$. *Inorg. Chem.* **2003**, 42 (19), 5857-5862. DOI: 10.1021/ic0342752

[11] Chen, W.; Liu, W.; Li, Y.; Wang, X.; Wang, E. Two new methylimidazole modified Hervé-sandwich-type polytungstoantimonates. *J. Coord. Chem*. **2011**, 64 (1), 71-81. DOI: 10.1080/00958972.2010.527003

[12] Grama, L.; Boda, F.; Gaz Florea, A. S.; Curticăpean, A.; Muntean, D. L. The UV and IR Com-parative Spectrophotometric Study of Some Saturated and Lacunary Polyoxometalates. *Acta Med. Marisiensis*. **2014**, 60 (3), 84-88. DOI: 10.2478/amma-2014-0017

[13] Patrut, A.; Bögge, H.; Forizs, E.; Rusu, D.; Lowy, D.; Margineanua, D.; Naumescue, A. Spec-troscopic and crystal structure investigation of a new bismuth (III) containing polyoxometalate cluster. *Rev. Roum. Chim.* **2010**, *55* (11-12), 865-870.

[14] Leifer, A.; Lippincott, E. R. The Infrared Spectra of Some Amino Acids. *J. Am. Chem. Soc.* **1957**, 79 (19), 5098-5101. DOI: 10.1021/ja01576a006

[15] Mueller, J. H.; Hinton, J. A Protein-Free Medium for Primary Isolation of the Gonococcus and Meningococcus. *Proc. Soc. Exp. Biol. Med.* **1941**, 48 (1), 330-333. DOI: 10.3181/00379727-48-13311
